# Distance-Based Knowledge Measure for Intuitionistic Fuzzy Sets with Its Application in Decision Making

**DOI:** 10.3390/e23091119

**Published:** 2021-08-28

**Authors:** Xuan Wu, Yafei Song, Yifei Wang

**Affiliations:** 1 School of Postgraduate School, Air Force Engineering University, Xi’an 710051, China; xuanwud@163.com; 2 School of Air and Missile Defense, Air Force Engineering University, Xi’an 710051, China

**Keywords:** Atanassov’s intuitionistic fuzzy sets, malicious code, distance measure, knowledge measure, uncertainty measure, decision making

## Abstract

Much attention has been paid to construct an applicable knowledge measure or uncertainty measure for Atanassov’s intuitionistic fuzzy set (AIFS). However, many of these measures were developed from intuitionistic fuzzy entropy, which cannot really reflect the knowledge amount associated with an AIFS well. Some knowledge measures were constructed based on the distinction between an AIFS and its complementary set, which may lead to information loss in decision making. In this paper, knowledge amount of an AIFS is quantified by calculating the distance from an AIFS to the AIFS with maximum uncertainty. Axiomatic properties for the definition of knowledge measure are extended to a more general level. Then the new knowledge measure is developed based on an intuitionistic fuzzy distance measure. The properties of the proposed distance-based knowledge measure are investigated based on mathematical analysis and numerical examples. The proposed knowledge measure is finally applied to solve the multi-attribute group decision-making (MAGDM) problem with intuitionistic fuzzy information. The new MAGDM method is used to evaluate the threat level of malicious code. Experimental results in malicious code threat evaluation demonstrate the effectiveness and validity of proposed method.

## 1. Introduction

Atanassov [1,2] developed the concept of intuitionistic fuzzy set on the basis of Zadeh’s fuzzy set [3]. Atanassov’s intuitionistic fuzzy sets (AIFSs) relax the condition that the non-membership degree and the membership degree sum to 1. AIFSs are a generalization of fuzzy sets, i.e., a particular case of other types of generalized fuzzy sets [4,5]. Moreover, AIFSs are identical to interval-valued fuzzy sets (IVFSs) from a mathematical perspective [6]. In an AIFS, the hesitation degree is the difference between one and the sum of membership and non-membership grades. The hesitation degree contributes much serviceability to the depiction of uncertain information. Researchers have paid much attention on the intuitionistic fuzzy set theory since its advantage in modeling uncertain information systems [7]. The theory of intuitionistic fuzzy sets has been successfully applied in many fields, including uncertainty reasoning [8] and decision making [9,10]. The connection between AIFSs and other uncertain theories is also attracting increasingly much interest [11,12,13,14,15,16,17,18].

Zadeh [3] first introduced the notion of entropy to fuzzy sets to measure the uncertainty or fuzziness in a fuzzy set. The notion of fuzzy entropy defined for fuzzy sets is partially similar to the concept of Shannon entropy [19], which was initially defined in probability theory. Luca and Termini [20] developed the axiomatic definition of entropy, and then proposed a kind of non-probabilistic fuzzy entropy. Then, Burillo and Bustince [21] first axiomatically defined the measure of intuitionistic entropy, which was merely determined by hesitation degree. Unlike the entropy measures created by Burillo and Bustince [21], the entropy measure for intuitionistic fuzzy sets developed by Szmidt and Kacprzyk [22] was defined based on the ratio of two distance values. Axiomatic definition for intuitionistic fuzzy entropy was also presented by Szmidt and Kacprzyk [22]. Following the work of Szmidt and Kacprzyk [22], many authors [23,24,25,26] have done a great deal of work concentrating on the definition of entropy measures. Some research has also focused on the entropy of AIFSs and their application in the evaluation of attribution weighting vector [9,10]. It has been pointed out by Szmidt et al. [27] that entropy measure cannot capture all uncertainty hidden in an AIFS. Thus, it may be difficult to develop a satisfactory uncertainty measure for AIFSs merely by entropy measure. The difference between entropy and hesitation in measuring the uncertainty of AIFSs has been pointed out by Pal et al. [28]. In [28], it was claimed that the combination of entropy and hesitation may furnish an effective way to measure the total uncertainty hidden in an AIFS.

Generally, knowledge measure is related to the useful information provided by an AIFS. From the perspective of information theory, much information indicates a great amount of knowledge, which is helpful for decision making. Therefore, the notion of knowledge measure can be regarded as the complementary concept of total uncertainty measure, rather than of entropy measure. This means that less total uncertainty always accompanies a greater amount of knowledge. With the purpose of making an evident distinction between types of intuitionistic fuzzy information, Szmidt et al. [27] took both intuitionistic fuzzy entropy and hesitation into consideration to develop a knowledge measure for AIFS, in which the intuitionistic fuzzy entropy was defined by quantifying the ration between the nearer distance and farer distance. This knowledge measure has been used to estimate the weight of each attribute to solve multi-attribute decision making (MADM) problems [29]. Nguyen [30] has developed a novel knowledge measure by measuring the distance from an AIFS to the most uncertain AIFS. It seems that this knowledge measure can well describe fuzziness and intuitionism in AIFSs. However, the use of normalized Euclidean distance may bring another problem, namely that the relation between fuzziness and knowledge cannot be completely reflected. Recently, Guo [29] put forward a new axiomatic definition for the knowledge measure of AIFS. A new and highly robust model was introduced in [31] to quantify the knowledge amount of AIFS. By measuring the difference between an AIFS and its complement, the new model proposed by Guo [31] has been widely used to defined entropy measure for AIFSs [32,33]. Moreover, the combination of the two parts in Guo’s model [31] lacks a clear physical interpretation. Several years ago, Das et al. [34] performed a comprehensive review of axiomatic definitions of information measures of AIFSs and investigated their relationships, in which entropy measure, knowledge measure, distance measure, and similarity measure are all concerned.

The above analysis demonstrates that the topic of knowledge measure for AIFSs is still open for debate, and commanding prodigious attention. Most research on knowledge and uncertainty measures of AIFSs mainly focus on the difference between AIFS and its complement. Only a few knowledge measures are constructed by measuring the distinction between an AIFS and the AIFS with maximum uncertainty or minimum uncertainty. Although Nguyen [30] opened up this new way of studying knowledge measures of AIFSs, further exploration is needed to improve this kind of knowledge measure and realize a desirable knowledge measure for AIFSs. This motivates us to present a new method to measure the knowledge of AIFSs based on a novel intuitionistic fuzzy distance, which is defined based on the transformation from an intuitionistic fuzzy value (IFV) to an interval value. An axiomatic definition of the knowledge measure of AIFSs will also be formulated from a more general point of view. Moreover, we will further explore the proposed knowledge measure’s properties, and we compare it with other measures based on numerical examples to demonstrate its performance. Then we will apply it to the problem of intuitionistic fuzzy multi-attribute group decision making (MAGDM).

The remainder of this study is structured as follows. Several concepts regarding AIFSs are explained in Section 2. In Section 3, a new type of distance measure for AIFSs is developed, followed by the proposal and discussion of the distance-based knowledge measure in Section 4. In Section 5, the proposed distance and knowledge measures are used to develop a new method to solve MAGDM problems in intuitionistic fuzzy condition. Application of the new method for MAGDM is presented in Section 6 to illustrate the performance of the proposed method. Some conclusions of this paper are presented in Section 7.

## 2. Preliminaries

Here, we briefly recount some background knowledge about AIFSs to for ease of subsequent exposition.

**Definition** **1.** *Letting a non-empty set* $X=\{x_{1},x_{2},\cdots ,x_{n}\}$*be the universe of discourse, a fuzzy set* A *in* X *is then defined as follows [3]:*

(1) $$A=\left\{\langle x,\mu_{A}(x)\rangle \left|x\in X\right.\right\}$$

*where*$\mu_{A}:X\to [0,1]$*is the membership degree.*

**Definition** **2.** *The intuitionistic fuzzy set* B*in* $X=\{x_{1},x_{2},\cdots ,x_{n}\}$ *as defined by Atanassov can be expressed as [1]:*

(2) $$B=\left\{\langle x,\mu_{B}(x),v_{B}(x)\rangle \left|x\in X\right.\right\}$$

*where*$\mu_{B}:X\to [0,1]$*and*$v_{B}:X\to [0,1]$*are membership degree and non-membership degree, respectively, with the condition*

(3) $$0\leq \mu_{B}(x)+v_{B}(x)\leq 1$$

*The hesitation degree of AIFS*B*defined in*X*is denoted*$\pi_{B}$. ∀x∈X*, and the hesitation degree is calculated by the expression that follows:*

(4) $$\pi_{B}(x)=1-\mu_{B}(x)-v_{B}(x)$$

Apparently, we can obtain $\pi_{B}(x)\in [0,1]$, ∀x∈X. $\pi_{B}(x)$ is also referred to as the intuitionistic index of x to B. Greater $\pi_{B}(x)$ indicates more vagueness. It is apparent that when $\pi_{B}(x)=0$, ∀x∈X, the AIFS degenerates into an ordinary fuzzy set.

For two AIFSs A and B defined in X, the following relations were defined in [1]: A⊇B if and only if $\mu_{A}(x)\geq \mu_{B}(x)$, $v_{A}(x)\leq v_{B}(x)$ for each x∈X. The complement of B is denoted $B^{C}$ [1], and can be obtained by $B^{C}=\left\{\langle x,v_{B}(x),\mu_{B}(x)\rangle \left|x\in X\right.\right\}$.

It has been proved that AIFSs and IVFSs are mathematically identical [4,6]. They can be converted to each other. Thus, For an AIFS B defined in X and x∈X, we can use an interval $\left[\mu_{B}(x),1-v_{B}(x)\right]$ to express the membership and non-membership grades of x with respect to B. We can see this as the interval-valued interpretation of AIFS, in which $\mu_{B}(x)$ and $1-v_{B}(x)$ represent the lower bound and upper bound of membership degree, respectively. Apparently, $\left[\mu_{B}(x),1-v_{B}(x)\right]$ is a valid interval, since $\mu_{B}(x)\leq 1-v_{B}(x)$ always holds for $\mu_{B}(x)+v_{B}(x)\leq 1$. The correspondence relation between AIFSs and IVFSs holds only from the mathematical point of view. If we explore their conceptual explanation and practical application, they may differ in the description of uncertainty [9,35].

In what follows, AIFSs(X) is used to denote the set consisted of all AIFSs defined in X. Generally, the couple $\langle \mu_{B}(x),v_{B}(x)\rangle$ is also called an IFV for clarity.

**Definition** **3.** *For two IFVs* $a=\langle \mu_{a},v_{a}\rangle$*and* $b=\langle \mu_{b},v_{b}\rangle$*, the partial order between them is defined as* $a\leq b\Leftrightarrow \mu_{a}\leq \mu_{b},v_{a}\geq v_{b}$ *[1]*.

For all IFVs, based on the partial ranking order, we can obtain the smallest IFV as $\langle 0,1\rangle$, denoted by **0**, and the largest IFV as $\langle 1,0\rangle$, denoted by **1**.

For a linear order of IFVs, to rank multiple IFVs, Chen and Tan [36] defined the score function of an IFV as $S(a)=\mu_{a}-v_{a}$. Following the concept of score function for IFVs, Hong and Choi [37] developed an accuracy function $H(a)=\mu_{a}+v_{a}$ to depict the accuracy of IFV $a=\langle \mu_{a},v_{a}\rangle$. Xu [38] then proposed a ranking-order relation between two IFVs a and b, which can be equivalently shown as follows.

For their score functions, if S(a) is greater than S(b), then a is greater than b, and vice versa.

If S(a) and S(b) are equal, we consider the following cases: (1) if H(a) is equal to H(b), then a and b are equal; and (2) if H(a) is greater than H(b), then a is greater than b; and vice versa.

Based on above order relation, the linear order relation of multiple IFVs can be obtained.

We know that similarity measure and distance measure are important in the research of fuzzy set theory [39]. Similarly, the construction of similarity measure and distance measures for AIFSs plays an important role in AIFSs [23,40,41,42,43,44,45,46,47,48,49,50], and they are helpful for the comparison of intuitionistic fuzzy information [24,25].

**Definition** **4.** *For a mapping* D:AIFS×AIFS→[0,1]*, it is called a distance measure between two AIFSs A and B defined in X if* D(A,B)*satisfies the following properties [23]:*

*(DP1)*0≤D(A,B)≤1*;*
*(DP2)*D(A,B)=0*, if and only if*A=B*;*
*(DP3)*D(A,B)=D(B,A)*;*
*(DP4) If*A⊆B⊆C*, then*D(A,B)≤D(A,C)*and*D(B,C)≤D(A,C)

**Definition** **5.** *Amapping* S:AIFS×AIFS→[0,1]*is called a similarity measure two AIFSs A and B defined in X if* S(a,b)*satisfies the following properties [40]:*

*(SP1)*0≤S(A,B)≤1*;*
*(SP2)*S(A,B)=1*, if and only if*A=B*;*
*(SP3)*S(A,B)=S(B,A)*;*
*(SP4) If*A⊆B⊆C*, then*S(A,B)≥S(A,C)*and*S(B,C)≥S(A,C).

Similarity measure and distance measure usually are regarded as a couple of dual concepts. Thus, distance measures can be used to define similarity measures, and vice versa.

## 3. New Intuitionistic Fuzzy Distance Measure

In past years, numerous similarity measure and distance measure have been advanced [7,39,45]. However, some may lead to unreasonable results in practical applications [7]. Some new defined distance/similarity measures may have complicated expressions [39,45], which are not suitable for constructing knowledge measure for AIFSs. Thus, it is necessary to define a desirable distance measure to assist us in developing a new knowledge measure. Here, we propose a new distance measure for AIFSs by borrowing a distance measure for interval values. It has been claimed that an AIFS can be represented in the form of interval-valued fuzzy set [5]. Based on such relation, an intuitionistic fuzzy distance measure can be developed based on interval comparison.

### 3.1. Interval-Comparison-Based Distance Measure for AIFSs

AnAIFS $B=\left\{\langle x,\mu_{B}(x),v_{B}(x)\rangle \left|x\in X\right.\right\}$ defined in $X=\{x_{1},x_{2},\cdots ,x_{n}\}$ indicates the membership degree of *x_i_* to *B* is uncertain, with lower and upper bounds of $\mu_{B}(x_{i})$ and $1-v_{B}(x_{i})$, respectively. That is to say, the membership grade of *x_i_* to *B* lies in an interval $\left[\mu_{B}(x_{i}),1-v_{B}(x_{i})\right]$, i=1,2,⋯,n. Thus, we can measure distance between AIFSs *A* and *B* defined in $X=\{x_{1},x_{2},\cdots ,x_{n}\}$ by comparing interval values $\left[\mu_{A}(x_{i}),1-v_{A}(x_{i})\right]$ and $\left[\mu_{B}(x_{i}),1-v_{B}(x_{i})\right]$, i=1,2,⋯,n.

In [51], authors have reviewed distances between interval values. They pointed out that the distance measure *d_TD_* proposed in [52] is not a metric distance, since for an interval value *a* = [*a*_1_,*a*_2_], *d_TD_*(*a*, *a*) = 0 does not always hold. Thus, Irpino and Verde [51] proposed a Wasserstein distance based on the point of view of one-dimensional uniform distribution, rather than from that of two-dimensional uniform distribution as developed in [52]. The definition as follows gives the Wasserstein distance measure between interval values.

**Definition** **6.** *Given two interval values a = [a_1_,a_2_] and b = [b_1_,b_2_] with* a,b∈[0,1]*, the distance between them is defined as [51]:*

(5) $$d^{I}(a,b)=\sqrt{{\left(\frac{a_{1}+a_{2}}{2}-\frac{b_{1}+b_{2}}{2}\right)}^{2}+\frac{1}{3}{\left(\frac{a_{2}-a_{1}}{2}-\frac{b_{2}-b_{1}}{2}\right)}^{2}}$$

*Thus,* ∀i∈{1,2,⋯,n} *, and for* $A_{x_{i}}=\langle \mu_{A}(x_{i}),v_{A}(x_{i})\rangle$ *and* $B_{x_{i}}=\langle \mu_{B}(x_{i}),v_{B}(x_{i})\rangle$ *the distance between their corresponding interval values* $\left[\mu_{A}(x_{i}),1-v_{A}(x_{i})\right]$ *and* $\left[\mu_{B}(x_{i}),1-v_{B}(x_{i})\right]$ *can be expressed by*

(6) $$d^{I}(A_{x_{i}},B_{x_{i}})=\sqrt{{\left(\frac{\mu_{A}(x_{i})+1-v_{A}(x_{i})}{2}-\frac{\mu_{B}(x_{i})+1-v_{B}(x_{i})}{2}\right)}^{2}+\frac{1}{3}{\left(\frac{1-v_{A}(x_{i})-\mu_{A}(x_{i})}{2}-\frac{1-v_{B}(x_{i})-\mu_{B}(x_{i})}{2}\right)}^{2}}$$

*which can also be expressed as*

(7) $$d^{I}(A_{x_{i}},B_{x_{i}})=\sqrt{{\left(\frac{\mu_{A}(x_{i})-v_{A}(x_{i})}{2}-\frac{\mu_{B}(x_{i})-v_{B}(x_{i})}{2}\right)}^{2}+\frac{1}{3}{\left(\frac{\mu_{A}(x_{i})+v_{A}(x_{i})}{2}-\frac{\mu_{B}(x_{i})+v_{B}(x_{i})}{2}\right)}^{2}}$$

*or*

(8) $$d^{I}(A_{x_{i}},B_{x_{i}})=\sqrt{{\left(\frac{\mu_{A}(x_{i})-v_{A}(x_{i})}{2}-\frac{\mu_{B}(x_{i})-v_{B}(x_{i})}{2}\right)}^{2}+\frac{1}{3}{\left(\frac{\pi_{A}(x_{i})}{2}-\frac{\pi_{B}(x_{i})}{2}\right)}^{2}}$$

*Since all parameters*$\mu_{A}(x_{i})$*,*$v_{A}(x_{i})$*,*$\pi_{A}(x_{i})$*,*$\mu_{B}(x_{i})$*,*$v_{B}(x_{i})$*, and*$\pi_{B}(x_{i})$*take values in the interval* [0,1]*, we have* $-1\leq \mu_{A}(x_{i})-v_{A}(x_{i})\leq 1$*,* $-1\leq \mu_{B}(x_{i})-v_{B}(x_{i})\leq 1$*. The maximum value of* $d^{I}(A_{x_{i}},B_{x_{i}})$ *can then be obtained as 1, which is obtained when* $A_{x_{i}}=\langle 0,1\rangle$*,* $B_{x_{i}}=\langle 1,0\rangle$ *or* $A_{x_{i}}=\langle 1,0\rangle$*,* $B_{x_{i}}=\langle 0,1\rangle$*. Thus, the relation* $0\leq d^{I}(A_{x_{i}},B_{x_{i}})\leq 1$ *can be obtained.*

According to the analysis above, we are able to define a new distance measure for Atanassov’s intuitionistic fuzzy sets. Given two AIFSs $A=\left\{\langle x,\mu_{A}(x),v_{A}(x)\rangle \left|x\in X\right.\right\}$ and $B=\left\{\langle x,\mu_{B}(x),v_{B}(x)\rangle \left|x\in X\right.\right\}$ defined in $X=\{x_{1},x_{2},\cdots ,x_{n}\}$, then the distance between them is calculated by the expression that follows:

(9) $$D^{I}(A,B)=\frac{1}{n}\sum_{i=1}^{n}\sqrt{{\left(\frac{\mu_{A}(x_{i})-v_{A}(x_{i})}{2}-\frac{\mu_{B}(x_{i})-v_{B}(x_{i})}{2}\right)}^{2}+\frac{1}{3}{\left(\frac{\mu_{A}(x_{i})+v_{A}(x_{i})}{2}-\frac{\mu_{B}(x_{i})+v_{B}(x_{i})}{2}\right)}^{2}}$$

**Theorem** **1.** *For AIFSs* $A=\left\{\langle x,\mu_{A}(x),v_{A}(x)\rangle \left|x\in X\right.\right\}$*and* $B=\left\{\langle x,\mu_{B}(x),v_{B}(x)\rangle \left|x\in X\right.\right\}$ *defined in* $X=\{x_{1},x_{2},\cdots ,x_{n}\}$, $D^{I}(A,B)$ *is a distance measure between A and B.*

For the sake of readability, we provide the proof process of Theorem 1 in Appendix A.

Considering the weight of $x_{i}$, i=1,2,⋯,n, the distance between AIFSs $A=\left\{\langle x,\mu_{A}(x),v_{A}(x)\rangle \left|x\in X\right.\right\}$ and $B=\left\{\langle x,\mu_{B}(x),v_{B}(x)\rangle \left|x\in X\right.\right\}$ defined in $X=\{x_{1},x_{2},\cdots ,x_{n}\}$ can be measured as

(10) $$D_{W}^{I}(A,B)=\sum_{i=1}^{n}w_{i}\sqrt{{\left(\frac{\mu_{A}(x_{i})-v_{A}(x_{i})}{2}-\frac{\mu_{B}(x_{i})-v_{B}(x_{i})}{2}\right)}^{2}+\frac{1}{3}{\left(\frac{\mu_{A}(x_{i})+v_{A}(x_{i})}{2}-\frac{\mu_{B}(x_{i})+v_{B}(x_{i})}{2}\right)}^{2}}$$

where $w_{i}$ is the weight of $x_{i}$, i=1,2,⋯,n, with $w_{i}\in [0,1]$ and $\sum_{i=1}^{n}w_{i}=1$.

**Theorem** **2.** $D_{W}^{I}(A,B)$*is distance measure between AIFSs* $A=\left\{\langle x,\mu_{A}(x),v_{A}(x)\rangle \left|x\in X\right.\right\}$ *and* $B=\left\{\langle x,\mu_{B}(x),v_{B}(x)\rangle \left|x\in X\right.\right\}$ *defined in* $X=\{x_{1},x_{2},\cdots ,x_{n}\}$.

Its proof can be implemented in the same way as the proof of Theorem 1.

### 3.2. Comparative Analysis

By way of demonstrating the availability of the new distance measure to distinguish the information in form of intuitionistic fuzzy set, we apply numerical examples to conduct a comparative analysis. Owing to the complementary relation between distance measure and similarity measure, the below widely used measures defined for two AIFSs $A=\left\{\langle x,\mu_{A}(x),v_{A}(x)\rangle \left|x\in X\right.\right\}$ and $B=\left\{\langle x,\mu_{B}(x),v_{B}(x)\rangle \left|x\in X\right.\right\}$ defined in $X=\{x_{1},x_{2},\cdots ,x_{n}\}$ will be used for comparison.

1. Hamming distance [53]:
  (11) $$D_{NH}(A,B)=\frac{1}{2n}\sum_{i=1}^{n}\left(\left|\mu_{A}(x_{i})-\mu_{B}(x_{i})\right|+\left|v_{A}(x_{i})-v_{B}(x_{i})\right|+\left|\pi_{A}(x_{i})-\pi_{B}(x_{i})\right|\right)$$
2. Euclidean distance [53]:
  (12) $$D_{NE}(A,B)=\sqrt{\frac{1}{2n}\sum_{i=1}^{n}\left({\left(\mu_{A}(x_{i})-\mu_{B}(x_{i})\right)}^{2}+{\left(v_{A}(x_{i})-v_{B}(x_{i})\right)}^{2}++{\left(\pi_{A}(x_{i})-\pi_{B}(x_{i})\right)}^{2}\right)}$$
3. Distance measurement of Wang and Xin [23]:
  (13) $$D_{W}(A,B)=\frac{1}{n}\sum_{i=1}^{n}\left[\frac{\left|\mu_{A}(x_{i})-\mu_{B}(x_{i})\right|+\left|v_{A}(x_{i})-v_{B}(x_{i})\right|}{4}\right.+\left.\frac{\max\left(\left|\mu_{A}(x_{i})-\mu_{B}(x_{i})\right|,\left|v_{A}(x_{i})-v_{B}(x_{i})\right|\right)}{2}\right]$$
4. Ye’s cosine similarity measure *C_IFS_* [54]:
  (14) $$C_{IFS}(A,B)=\frac{1}{n}\sum_{i=1}^{n}\frac{\mu_{A}(x_{i})\mu_{B}(x_{i})+v_{A}(x_{i})v_{B}(x_{i})}{\sqrt{{\left(\mu_{A}(x_{i})\right)}^{2}+{\left(v_{A}(x_{i})\right)}^{2}}\sqrt{{\left(\mu_{B}(x_{i})\right)}^{2}+{\left(v_{B}(x_{i})\right)}^{2}}}$$

**Example** **1.** *Three patterns are presented by AIFSs defined in* $X=\{x_{1},x_{2},x_{3}\}$*and are given as*

$$A_{1}=\{<x_{1},0.4,0.5>,<x_{2},0.7,0.1>,<x_{3},0.3,0.3>\},\;A_{2}=\{<x_{1},0.5,0.4>,<x_{2},0.7,0.2>,<x_{3},0.4,0.3>\},\;A_{3}=\{<x_{1},0.4,0.5>,<x_{2},0.7,0.1>,<x_{3},0.4,0.3>\}.$$

*A sample*$B=\{<x_{1},0.1,0.1>,<x_{2},1,0>,<x_{3},0,1>\}$*is given to be classified*.

Using Equations (11) and (12), we obtain

*D_NH_*(*A*_1_,*B*) = *D_NH_*(*A*_2_,*B*) = *D_NH_*(*A*_3_,*B*) = 0.483,

*D_NE_*(*A*_1_,*B*) = *D_NE_*(*A*_2_,*B*) = *D_NE_*(*A*_3_,*B*) = 0.442.

Using the proposed distance measure *D^I^*(*A*,*B*), we obtain

*D^I^*(*A*_1_,*B*) = 0.3098, *D^I^*(*A*_2_,*B*) = 0.3389, *D^I^*(*A*_3_,*B*) = 0.3244.

We note in this example that the Hamming and Euclidean distances cannot be used to determine the pattern of *B*. The new proposed measure *D^I^* can classify *B* as pattern *A*_1_ because the distance between *B* and *A*_1_ is the least.

**Example** **2.** *Three patterns are presented by AIFSs defined in* $X=\{x_{1},x_{2},x_{3},x_{4}\}$*and are given as*

$$A_{1}=\{<x_{1},0.3,0.4>,<x_{2},0.3,0.4>,<x_{3},0.6,0.1>,<x_{4},0.6,0.1>\},\;A_{2}=\{<x_{1},0.4,0.4>,<x_{2},0.3,0.5>,<x_{3},0.7,0.1>,<x_{4},0.6,0.2>\},\;A_{3}=\{<x_{1},0.4,0.4>,<x_{2},0.3,0.4>,<x_{3},0.7,0.1>,<x_{4},0.6,0.1>\}.$$

A sample to be classified is given as

$$B=\{<x_{1},0.35,0.65>,<x_{2},0.55,0.45>,<x_{3},0.65,0.1>,<x_{4},0.6,0.15>\}.$$

Using Equation (13), we can obtain: *D_W_*(*A*_1_,*B*) = *D_W_*(*A*_2_,*B*) = *D_W_*(*A*_3_,*B*) = 0.119.

Using our proposed distance measure *D^I^*(*A*,*B*), we obtain

*D^I^*(*A*_1_,*B*) = 0.0806, *D^I^*(*A*_2_,*B*) = 0.0948, *D^I^*(*A*_3_,*B*) = 0.0877.

These results show that the class of *B* cannot be determined based on the distance measure proposed by Wang and Xin [23]. Based on our proposed distance measure, we are able to obtain the minimum distance between B and three patterns as *D_I_*(*A*_1_,*B*) = 0.0806; therefore, sample B is classified to pattern *A*_1_.

**Example** **3.** *Three patterns expressed by AIFSs which are defined in* $X=\{x_{1},x_{2}\}$*are given as*

$$A_{1}=\{<x_{1},0.4,0.4>,<x_{2},0.3,0.3>\},\;A_{2}=\{<x_{1},0.2,0.2>,<x_{2},0.3,0.3>\},\;A_{3}=\{<x_{1},0.1,0.1>,<x_{2},0.5,0.5>\}.$$

An unknown sample to be recognized is given by

$$B=\{<x_{1},0.1,0.1>,<x_{2},0.5,0.5>\}.$$

Using Equation (14), we can get: *C_IFS_*(*A*_1_,*B*) = *C_IFS_*(*A*_2_,*B*) = *C_IFS_*(*A*_3_,*B*) = 1.

Using the proposed distance measure *D^I^*(*A*,*B*), we obtain:

*D^I^*(*A*_1_,*B*) = 0.1443, *D^I^*(*A*_2_,*B*) = 0.0866, *D^I^*(*A*_3_,*B*) = 0.

It is obvious that sample *B* is identical to pattern *A*_3_, but sample *B* may be classified as *A*_1_, *A*_2_, and *A*_3_ simultaneously based on the cosine similarity, which is counter-intuitive. It can be seen that our distance measure can be used in classifying sample *B* as *A*_3_ due to the zero distance between them.

The above examples show that our proposed distance measure is effective in differentiating the information conveyed by different AIFSs. It can be easily proved that the choice of attribute weights will not change the conclusion obtained based on each example. Moreover, we note that the cosine similarity may be undefined when there is a zero denominator. The developed distance measures can overcome such deficiencies, so these examples indicate that the proposed distance measures are reasonable and effective in discriminating intuitionistic fuzzy information.

## 4. Knowledge Measure of AIFSs Based on *D^I^*

Suppose that $A=\left\{\langle x,\mu_{A}(x),v_{A}(x)\rangle \left|x\in X\right.\right\}$ is an AIFS defined in $X=\{x_{1},x_{2},\cdots ,x_{n}\}$, its knowledge measure *K* should intuitively satisfy some properties. It is rational that the knowledge measure *K* must be a non-negative function determined by $\mu_{A}(x)$ and $v_{A}(x)$. The knowledge amount of *A* should be identical to the knowledge amount of its complement, i.e., *K*(*A*) = *K*(*A^C^*). When the AIFS *A* is reduced to classical Zadeh’s fuzzy set, a negative correlation should exist between the knowledge measure and fuzziness. It has been in our mind that the fuzziness of Zadeh’s fuzzy set determines its fuzzy entropy, and they are both negatively correlated to $\left|\mu_{A}(x)-v_{A}(x)\right|$ [22]. So the knowledge measure *K*(*A*) should be monotonously increasing with respect to $\left|\mu_{A}(x)-v_{A}(x)\right|$. Moreover, we note that a crisp set provides the maximum amount of information, so the knowledge amount of a crisp set reaches the maximum value $K_{\max}=1$. Conversely, the case that ∀x∈X, $\mu_{A}(x)=v_{A}(x)=0$ means full ignorance, so the knowledge amount reaches its minimum value $K_{\min}=0$. In addition, in the case of $\mu_{A}(x_{i})=v_{A}(x_{i})=a\neq 0$, we have $\pi_{A}(x_{i})=1-2a$. Thus, the less *a* indicates greater the greater hesitant degree $\pi_{A}(x_{i})$, which leads to the greater uncertainty degree and smaller knowledge amount.

Considering these intuitive properties, we give the following definition to describe the axiomatic properties of the knowledge measure for AIFSs.

**Definition** **7.** *If a mapping* K:AIFS→[0,1]*satisfies the following properties, it is called a knowledge measure of an AIFS A defined in* $X=\{x_{1},x_{2},\cdots ,x_{n}\}$*:*

*(KP1)*K(A)=1*if and only if A is a crisp set.*
*(KP2)*K(A)=0*if and only if*$\pi_{A}(x_{i})=1$*,*∀i∈{1,2,⋯,n}.
*(KP3)*K(A)*increases with*$\left|\mu_{A}(x_{i})-v_{A}(x_{i})\right|$*for fixed*$\pi_{A}(x_{i})$*and decreases with*$\pi_{A}(x_{i})$*if*$\left|\mu_{A}(x_{i})-v_{A}(x_{i})\right|$*is unchanged,*i=1,2,⋯,n.
*(KP4)*$K(A^{C})=K(A)$.

Since both knowledge and entropy measures are always regarded as two complementary concepts, we discuss these properties by comparing them with those of entropy measure. We can see that the third property in [22] defined for intuitionistic fuzzy entropy, denoted as *E*, is stated as: E(B)≤E(A) if *B* is less fuzzy than *A*, i.e., ∀x∈X, (1) $\mu_{B}(x)\leq \mu_{A}(x)$ and $v_{B}(x)\geq v_{A}(x)$ for $\mu_{A}(x)\leq v_{A}(x)$, or (2) $\mu_{B}(x)\geq \mu_{A}(x)$ and $v_{B}(x)\leq v_{A}(x)$ for $\mu_{A}(x)\geq v_{A}(x)$.

The first condition indicates that $\mu_{B}(x_{i})\leq \mu_{A}(x_{i})\leq v_{A}(x_{i})\leq v_{B}(x_{i})$ and $\left|\mu_{B}(x_{i})-v_{B}(x_{i})\right|$$\geq \left|\mu_{A}(x_{i})-v_{A}(x_{i})\right|$. Similarly, the second condition implies that $\mu_{B}(x_{i})\geq \mu_{A}(x_{i})$$\geq v_{A}(x_{i})\geq v_{B}(x_{i})$ and $\left|\mu_{B}(x_{i})-v_{B}(x_{i})\right|$$\geq \left|\mu_{A}(x_{i})-v_{A}(x_{i})\right|$. Therefore, the entropy measure of AIFS decreases with $\left|\mu_{A}(x_{i})-v_{A}(x_{i})\right|$, i.e., E(B)≤E(A) if $\left|\mu_{B}(x_{i})-v_{B}(x_{i})\right|\geq \left|\mu_{A}(x_{i})-v_{A}(x_{i})\right|$, i=1,2,⋯,n, which is related to the property of KP3. However, this property of intuitionistic fuzzy entropy does not consider the influence of hesitation degree. It may not be sensible to discuss the relationship between fuzziness and intuitionistic fuzzy entropy if the hesitance degree is not fixed. Moreover, since $\left|\mu_{A}(x_{i})-v_{A}(x_{i})\right|\geq \left|\mu_{B}(x_{i})-v_{B}(x_{i})\right|$ cannot always induce $\mu_{A}(x_{i})\leq \mu_{B}(x_{i})\leq v_{B}(x_{i})\leq v_{A}(x_{i})$ or $\mu_{A}(x_{i})\geq \mu_{B}(x_{i})\geq v_{B}(x_{i})\geq v_{A}(x_{i})$, the property E(A)≤E(B) if $\left|\mu_{A}(x_{i})-v_{A}(x_{i})\right|\geq \left|\mu_{B}(x_{i})-v_{B}(x_{i})\right|$ is more general than the third property listed in [22]. Thus, for the relation between knowledge and fuzziness, our proposed axiomatic property is made more general by relaxing the formal constraint by using $\left|\mu (x)-v(x)\right|$. However, such relaxation does not cause an unreliable measure of the knowledge amount because of the limitation of hesitation degree, which will be illustrated later. This also demonstrates the possibility and reasonability of further exploring the relation between the entropy measure and knowledge measure of AIFSs. We point out that the entropy of an AIFS reaches its peak value when the membership degree and non-membership degree are identical for all elements [22]. This is analogous to the entropy measure of fuzzy sets, which solely concerns the relation between membership degree and non-membership degree. Therefore, the notions of entropy and knowledge measure are not just complementary concepts, but rather they differ from each other not only in the aspect of viewpoint, but also in the point they focus on. The fuzzy entropy merely depicts the difference between an AIFS and a crisp set, which is denoted as fuzziness, while knowledge measure is defined to measure the closeness between AIFS and a crisp set, which takes both fuzziness and hesitancy into account.

Following the axiomatic properties in Definition 7, we can create knowledge measures for AIFSs by a mapping *F*: *D*→[0,1], where D={(x,y)∈[0,1]×[0,1]|x+y≤1}, and *F* must satisfy the following conditions:

(C1) *F*(*x*, *y*) = 1 if and only if |*x−y*| = 1.
(C2) *F*(*x*, *y*) = 0 if and only if *x* = *y* = 0.
(C3) For a fixed *x*+*y*, *F*(*x*, *y*) increases while |*x−y*| increases.
(C4) For a fixes |*x−y*|, *F*(*x*, *y*) increases while *x*+*y* increases.
(C5) *F*(*x*, *y*) = *F*(*y*, *x*).

For (x,y)∈[0,1]×[0,1], we can effortlessly obtain many *F* functions satisfying the above conditions, such as *F*(*x*,*y*) = (|*x−y*|+*x*+*y*)/2 and *F*(*x*,*y*) = *x*^2^+*y*^2^. Using these functions, we can construct knowledge measures for AIFSs. Given an AIFS $A=\left\{\langle x,\mu_{A}(x),v_{A}(x)\rangle \left|x\in X\right.\right\}$ defined in $X=\{x_{1},x_{2},\cdots ,x_{n}\}$, its knowledge measure *K* can be expressed by $K=\sum_{i=1}^{n}F(\mu_{A}(x_{i}),v_{A}(x_{i}))/n$. In this way, many knowledge measures can be created for AIFSs, but most may lack of specific physical meaning. This motivates us to construct knowledge measures with both clear physical significance and axiomatic mathematical properties.

### 4.1. Construction of Knowledge Measure

From the second property KP2, we can conclude that the AIFS $F=\{\langle x,0,0\rangle |x\in X\}$ conveys the least knowledge. The amount of knowledge conveyed by an AIFS *A* can be reflected by the distance between *A* and *F*. The greater the distance between them, the greater the knowledge amount the AIFS *A* conveys, prompting us to devise a knowledge measure according to the distance from *A* to *F*.

For an AIFS $A=\left\{\langle \mu_{A}(x),v_{A}(x)\rangle \right\}$ defined in X={x}, the distance between A and $F=\left\{\langle x,0,0\rangle \right\}$ can be calculated by Equation (9):

(15) $$D^{I}(A,F)=\sqrt{{\left(\frac{\mu_{A}(x)-v_{A}(x)}{2}\right)}^{2}+\frac{1}{3}{\left(\frac{\mu_{A}(x)+v_{A}(x)}{2}\right)}^{2}}$$

Equation (15) can be further written as

(16) $$D^{I}(A,F)=\frac{1}{2}\sqrt{{\left(\mu_{A}(x)-v_{A}(x)\right)}^{2}+\frac{1}{3}{\left(\mu_{A}(x)+v_{A}(x)\right)}^{2}}$$

Considering the conditions $0\leq \mu_{A}(x)\leq 1$, $0\leq v_{A}(x)\leq 1$, and $0\leq \mu_{A}(x)+v_{A}(x)\leq 1$, we have $-1\leq \mu_{A}(x)-v_{A}(x)\leq 1$. Since the conditions $\mu_{A}(x)+v_{A}(x)=1$ and $\left|\mu_{A}(x)-v_{A}(x)\right|=1$ can be satisfied simultaneously, the maximum value of $D^{I}(A,F)$ is $\sqrt{3}/3$. Thus, the distance between A and F can be normalized by multiplying by $\sqrt{3}$, giving the following form:

(17) $$D_{{}^{N}}^{I}(A,F)=\frac{\sqrt{3}}{2}\sqrt{{\left(\mu_{A}(x)-v_{A}(x)\right)}^{2}+\frac{1}{3}{\left(\mu_{A}(x)+v_{A}(x)\right)}^{2}}$$

We can then construct a knowledge measure for AIFSs defined in the discourse universe X={x} as follows:

(18) $$K^{I}(A)=\frac{\sqrt{3}}{2}\sqrt{{\left(\mu_{A}(x)-v_{A}(x)\right)}^{2}+\frac{1}{3}{\left(\mu_{A}(x)+v_{A}(x)\right)}^{2}}$$

Generally, for the AIFS defined in $X=\{x_{1},x_{2},\cdots ,x_{n}\}$, denoted as $A=\left\{\langle x,\mu_{A}(x),v_{A}(x)\rangle \left|x\in X\right.\right\}$, its knowledge amount can be quantified by

(19) $$K^{I}(A)=\frac{\sqrt{3}}{2n}\sum_{i=1}^{n}\sqrt{{\left(\mu_{A}(x_{i})-v_{A}(x_{i})\right)}^{2}+\frac{1}{3}{\left(\mu_{A}(x_{i})+v_{A}(x_{i})\right)}^{2}}$$

**Theorem** **3.** *For the AIFS* $A=\left\{\langle x,\mu_{A}(x),v_{A}(x)\rangle \left|x\in X\right.\right\}$*defined in* $X=\{x_{1},x_{2},\cdots ,x_{n}\}$*, the function* $K^{I}(A)$ *defined by Equation (19) is a knowledge measure of AIFS* A.

Theorem 3 is proved in Appendix B.

### 4.2. Numerical Examples

Here, the performance of the proposed knowledge measure *K^I^* will be examined considering some numerical examples.

**Example** **4.** *Four AIFSs A_1_, A_2_, A_3_ and A_4_ are defined in universe X = {x}. They are given as*

$$A_{1}=\left\{\langle x,0.5,0.5\rangle \right\},\;A_{2}=\left\{\langle x,0.3,0.3\rangle \right\},\;A_{3}=\left\{\langle x,0.2,0.2\rangle \right\},\;A_{4}=\left\{\langle x,0,0\rangle \right\}.$$

The entropy measure presented in [23,55,56,57,58] cannot discriminate these AIFSs, since these measures are defined according to the difference between membership degree and non-membership degree. The membership degree and non-membership degree are identical in these four AIFSs, so they may be considered identically with the maximal entropy, which induces a minimal knowledge amount conveyed by them. However, according to the proposed knowledge measure *K_I_*, we have

$$K^{I}(A_{1})=0.5,\;K^{I}(A_{2})=0.3,\;K^{I}(A_{3})=0.2,\;K^{I}(A_{4})=0$$

It can be seen that these four different AIFSs differ greatly from each other from the viewpoint of knowledge amount. This is helpful for handling such extreme cases with identical supporting and opposing degrees. From the definition of *K_I_*, we find that, when $\mu_{A}(x)=v_{A}(x)$ and for all x∈X, the calculation of *K_I_* assumes the following form:

(20) $$K^{I}(A)=\frac{1}{n}\sum_{i=1}^{n}\mu_{A}(x_{i})$$

which indicates that the knowledge amount increases with the variable $\mu_{A}(x_{i})$ in the conditions of $\mu_{A}(x_{i})=v_{A}(x_{i})$ and $\forall i\in \left\{1,2,\cdots ,n\right\}$. This useful feature coincides with intuitive analysis.

**Figure 1:**
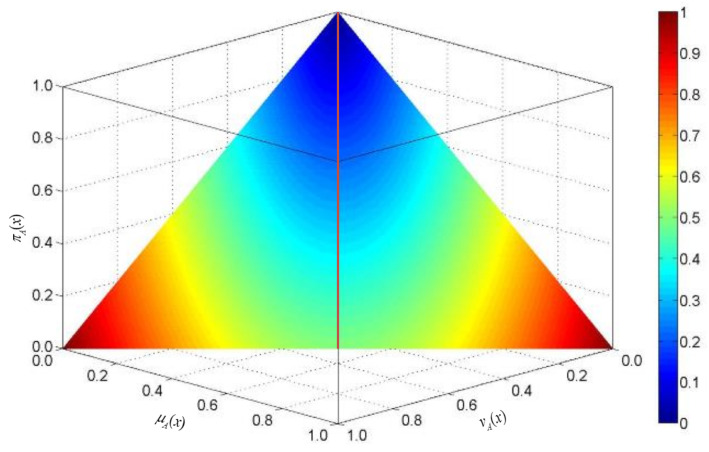
Knowledge amount KI of AIFSs defined in X={x}.

To further demonstrate the discriminability of the knowledge measure *K^I^*, we give Figure 1 to depict the value of knowledge amount associated with AIFS *A* defined in *X* = {*x*}. The value of *K^I^* (*A*) is reflected by the color assigned on each point $\left(\mu_{A}(x),v_{A}(x)\right)$ in the simplex. It is shown that the figure is symmetric along the line $\mu_{A}(x)=v_{A}(x)$, which illustrates the property of *K^I^* (*A^C^*) = *K^I^* (*A*). On the symmetric line $\mu_{A}(x)=v_{A}(x)$, the rising trend of knowledge amount is clear. As shown in Figure 1, the maximum amount of knowledge is obtained in two points, (0,1) and (1,0), and in the point (0,0) the knowledge amount is minimum.

**Example** **5.** *Let X = {6,7,8,9,10} be the discourse universe, an AIFS A in X is defined as:*

$$A=\left\{\langle 6,0.1,0.8\rangle ,\langle 7,0.3,0.5\rangle ,\langle 8,0.5,0.4\rangle ,\langle 9,0.9,0\rangle ,\langle 10,1,0\rangle \right\}.$$

De et al. [59] defined an exponent operation for AIFS A defined in X. Given a non-negative real number *m*, $A^{m}$ is defined as

(21) $$A^{m}=\left\{\left.\langle x,{\left(\mu_{A}(x)\right)}^{m},1-{\left(1-v_{A}(x)\right)}^{m}\rangle x\in X\right|\right\}$$

Based on the operations in Equation (21), we have

$$A^{0.5}=\left\{\langle 6,0.316,0.553\rangle ,\langle 7,0.548,0.293\rangle ,\langle 8,0.707,0.225\rangle ,\langle 9,0.949,0\rangle ,\langle 10,1,0\rangle \right\},\;A^{2}=\left\{\langle 6,0.010,0.960\rangle ,\langle 7,0.090,0.750\rangle ,\langle 8,0.250,0.640\rangle ,\langle 9,0.810,0\rangle ,\langle 10,1,0\rangle \right\},\;A^{3}=\left\{\langle 6,0.001,0.992\rangle ,\langle 7,0.027,0.875\rangle ,\langle 8,0.125,0.784\rangle ,\langle 9,0.729,0\rangle ,\langle 10,1,0\rangle \right\},\;A^{4}=\left\{\langle 6,0.0001,0.998\rangle ,\langle 7,0.008,0.938\rangle ,\langle 8,0.062,0.870\rangle ,\langle 9,0.656,0\rangle ,\langle 10,1,0\rangle \right\}.$$

Considering the characterization analysis of linguistic variables, we can consider AIFS *A* as “LARGE” in *X*. Correspondingly, AIFSs *A*^0.5^, *A*^2^, *A*^3^, and *A*^4^ can be regarded as “More or less LARGE,” “Very LARGE,” “Quite very LARGE,” and “Very LARGE,” respectively.

Intuitively, from *A*^0.5^ to *A*^4^, the uncertainty hidden in them becomes less and the knowledge amount conveyed by them increases. Therefore, the following relations hold:

(22) $$E\left(A^{0.5}\right)>E\left(A\right)>E\left(A^{2}\right)>E\left(A^{3}\right)>E\left(A^{4}\right)$$

(23) $$K\left(A^{0.5}\right)<K\left(A\right)<K\left(A^{2}\right)<K\left(A^{3}\right)<K\left(A^{4}\right)$$

To make a comparison, the entropy and knowledge measures listed in Table 1 are used. It is worth nothing that some of the entropy measures in the table are initially designed for interval valued fuzzy sets [56,57]. These entropy measures are modified for AIFSs based on their connection with interval values fuzzy sets. We present the results obtained based on different measures in Table 2 to facilitate comparative analysis.

From Table 2, we can see that entropy measures *E_ZL_*, *E_ZB_*, *E_BB_*, *E_SK_*, *E_HC_*, *E_S_*, and *E_ZJ_* induce the following relations:

$$E_{ZL}(A)>E_{ZL}(A^{0.5})>E_{ZL}(A^{2})>E_{ZL}(A^{3})>E_{ZL}(A^{4}),\;E_{ZB}(A)>E_{ZB}(A^{0.5})>E_{ZB}(A^{2})>E_{ZB}(A^{3})>E_{ZB}(A^{4}),\;E_{BB}(A)>E_{BB}(A^{0.5})>E_{BB}(A^{2})>E_{BB}(A^{3})=E_{BB}(A^{4}),\;E_{SK}(A)>E_{SK}(A^{0.5})>E_{SK}(A^{2})>E_{SK}(A^{3})>E_{SK}(A^{4}),\;E_{HC}(A)>E_{HC}(A^{0.5})>E_{HC}(A^{2})>E_{HC}(A^{3})>E_{HC}(A^{4}),\;E_{S}(A)>E_{S}(A^{0.5})>E_{S}(A^{2})>E_{S}(A^{3})>E_{S}(A^{4}),\;E_{ZJ}(A)>E_{ZJ}(A^{0.5})>E_{ZJ}(A^{2})>E_{ZJ}(A^{3})>E_{ZJ}(A^{4}).$$

Because the entropy of AIFS *A*^0.5^ is less than that of AIFS *A*, entropy measures *E_ZL_*, *E_ZB_*, *E_ZE_*, *E_BB_*, *E_SK_*, and *E_ZJ_* do not perform as well as other entropy measures. From the point of view of knowledge amount, we note that the results obtained by *K_SKB_*, *K_N_*, and *K_G_* are not so reasonable, since counter-intuitive relations $K_{SKB}(A^{0.5})>K_{SKB}(A)$, $K_{N}(A^{0.5})>K_{N}(A)$, and $K_{G}(A^{0.5})>K_{G}(A)$ exist. However, our developed knowledge measure *K^I^* can produce a rational result as *K^I^*(*A*^0.5^) < *K^I^*(*A*) < *K^I^*(*A*^2^) < *K^I^*(*A*^3^) *< K^I^*(*A*^4^). Thus, it is demonstrated that half of entropy measures in Table 1 cannot reflect the uncertainty hidden in these AIFSs. Although several knowledge measures have been presented, they are not able to distinguish the nuance of knowledge amount in different AIFSs. Thus, our developed knowledge measure outperforms other knowledge measures by providing persuasive results complying with intuitive analysis.

For a further investigation of the performance of the proposed knowledge measure, we modify the AIFS “LARGE” defined in *X* = {6,7,8,9,10} by increasing the non-membership degree of element “8” and reducing its hesitant degree. The modified AIFS “LARGE” is given as

$$B=\left\{\langle 6,0.1,0.8\rangle ,\langle 7,0.3,0.5\rangle ,\langle 8,0.5,0.5\rangle ,\langle 9,0.9,0\rangle ,\langle 10,1,0\rangle \right\}.$$

Through the operation shown in Equation (21), the following AIFSs related to *B* can be generated:

$$B^{0.5}=\left\{\langle 6,0.316,0.553\rangle ,\langle 7,0.548,0.293\rangle ,\langle 8,0.707,0.293\rangle ,\langle 9,0.949,0\rangle ,\langle 10,1,0\rangle \right\},\;B^{2}=\left\{\langle 6,0.010,0.960\rangle ,\langle 7,0.090,0.750\rangle ,\langle 8,0.250,0.750\rangle ,\langle 9,0.810,0\rangle ,\langle 10,1,0\rangle \right\},\;B^{3}=\left\{\langle 6,0.001,0.992\rangle ,\langle 7,0.027,0.875\rangle ,\langle 8,0.125,0.875\rangle ,\langle 9,0.729,0\rangle ,\langle 10,1,0\rangle \right\},\;B^{4}=\left\{\langle 6,0.0001,0.998\rangle ,\langle 7,0.008,0.938\rangle ,\langle 8,0.062,0.938\rangle ,\langle 9,0.656,0\rangle ,\langle 10,1,0\rangle \right\}.$$

According to the entropy and knowledge measures listed in Table 1, we obtain the comparative results as shown in Table 3.

It can be seen that AIFS *B* still has more entropy than AIFS *B*^0.5^ when entropy measures *E_ZL_*, *E_ZB_*, *E_ZE_*, *E_BB_*, *E_SK_*, and *E_ZJ_* are considered. The ordered results obtained based on these entropy measures are

$$E_{ZL}(B)>E_{ZL}(B^{0.5})>E_{ZL}(B^{2})>E_{ZL}(B^{3})>E_{ZL}(B^{4}),\;E_{ZB}(B)>E_{ZB}(B^{0.5})>E_{ZB}(B^{2})>E_{ZB}(B^{3})>E_{ZB}(B^{4}),\;E_{ZE}(B)>E_{ZE}(B^{0.5})>E_{ZE}(B^{2})>E_{ZE}(B^{3})>E_{ZE}(B^{4}),\;E_{BB}(B)>E_{BB}(B^{0.5})>E_{BB}(B^{2})>E_{BB}(B^{3})>E_{BB}(B^{4}),\;E_{SK}(B)>E_{SK}(B^{0.5})>E_{SK}(B^{2})>E_{SK}(B^{3})>E_{SK}(B^{4}),\;E_{ZJ}(B)>E_{ZJ}(B^{0.5})>E_{ZJ}(B^{2})>E_{ZJ}(B^{3})>E_{ZJ}(B^{4}).$$

It can be seen that these ranked orders do not satisfy intuitive analysis in Equation (22), while other entropy measures can induce desirable results. In this example, *E_HC_* and *E_S_* perform well, but the measure *E_ZE_* performs poorly. This illustrates that these entropy measures are not robust enough.

Moreover, the results produced by knowledge measures *K_SVB_*, *K_N_*, and *K_G_* are also not reasonable, shown as:

$$K_{SVB}(B)<K_{SVB}(B^{0.5})<K_{SVB}(B^{2})<K_{SVB}(B^{3})<K_{SVB}(B^{4}),\;K_{N}(B)<K_{N}(B^{0.5})<K_{N}(B^{2})<K_{N}(B^{3})<K_{N}(B^{4}),\;K_{G}(B)<K_{G}(B^{0.5})<K_{G}(B^{2})<K_{G}(B^{3})<K_{G}(B^{4}).$$

However, our proposed knowledge measure *K^I^* indicates that:

$$K^{I}(B^{0.5})<K^{I}(B)<K^{I}(B^{2})<K^{I}(B^{3})<K^{I}(B^{4}).$$

Thus, the knowledge measures *K_SVB_*, *K_N_*, and *K_G_* are still not suitable for differentiating the knowledge amount conveyed by AIFSs. The effectiveness of the proposed knowledge measure *K^I^* is once again indicated by this example.

From the above examples, we conclude that entropy measures *E_ZL_*, *E_ZB_*, *E_ZE_*, *E_BB_*, *E_HC_*, *E_S_*, *E_SK_*, and *E_ZJ_* perform poorly because of their lack of robustness and discriminability. The proposed knowledge measure performs much better than knowledge measures *K_SVB_*, *K_N_*, and *K_G_*. The performances of entropy measures *E_A_*, *E_ZC_*, *E_ZD_*, *E_VS_*, *E_LDL_*, and the proposed knowledge measure *K^I^* in Table 3 seem to show that less entropy indicates more knowledge amount. Nevertheless, the relationship between entropy and knowledge measure is limited and conditional, as was discussed previously.

The above analysis indicates an effective way to define knowledge measure for AIFSs based on a metric distance measure *d*_AIFS_ for AIFSs.

## 5. New Method for Solving MAGDM Problems

Since the inception of AIFSs, many researchers have been dedicated to exploring applications of AIFSs along with their mathematical mechanism. One important application area of AIFSs is multi-attribute group decision making (MAGDM) [28,30,36,38,62,63]. In the MAGDM problem, because of the limitation of experts’ knowledge and time pressure, uncertain or incomplete information may be provided in the evaluation of each alternative. Therefore, a suitable model should be constructed to depict the incomplete information. By introducing hesitancy degree, AIFSs can describe the uncertainty caused both by fuzziness and by lack of knowledge. Moreover, incomplete information can be aggregated in a direct way with the help of intuitionistic fuzzy aggregation operators. Thus, AIFSs are accepted by many researchers as one effective tool for solving MAGDM problems. The application of AIFSs in solving MAGDM problems has attract many researchers because of a series of open topics in this area, such as the determination of attribute weights, effective aggregation operators for AIFSs, ranking of alternatives based on IFVs, and the construction of intuitionistic fuzzy model from incomplete information.

Here, we put forth a new method with which to solve intuitionistic fuzzy MAGDM problems. We develop the approach according to the proposed intuitionistic fuzzy distance measure and distance-based knowledge measure. The intuitionistic fuzzy MAGDM problem is depicted as follows.

$G=\{G_{1},G_{2},\cdots ,G_{m}\}$ is the set consisted of all threat levels. $A=\{A_{1},A_{2},\cdots ,A_{n}\}$ is the set containing all attributes which will be considered to evaluate the threat level. $E=\{E_{1},E_{2},\cdots ,E_{s}\}$ is the set of all decision makers to evaluate threat levels. The weight of attribute *A_i_* is *w_i_*, i=1,2,⋯,n, with $\sum_{i=1}^{n}w_{i}=1$. All weights are expressed by weight vector $w={\left(w_{1},w_{2},\cdots ,w_{n}\right)}^{T}$. Each decision maker is assigned a weighting factor $\lambda_{j}$, j=1,2,⋯,s, with $\sum_{j=1}^{s}\lambda_{j}=1$. Decision maker *E_k_* (k=1,2,⋯,s) gives the decision matrix expressed by IFVs as:

(24) $$\begin{array}{l} \;\;\;\;\;\;\;\;\;\;\;\;\;\;\;\;\begin{array}{cccc} \;\;\;\;\;\;\;\;\;\;\;\;\;\;\;\;\;\;A_{1} & \;\;\;\;\;\;\;\;\;\;\;\;\;\;A_{2} & \;\;\;\;\cdots & \;\;\;\;\;\;\;\;A_{n} \end{array}\\ R_{k}=\;\;\;\begin{array}{l} G_{1}\\ G_{2}\\ \;\;\vdots \\ G_{m} \end{array}\;\;\;\;\left(\begin{array}{cccc} \langle \mu_{11}^{k},v_{11}^{k}\rangle & \langle \mu_{12}^{k},v_{12}^{k}\rangle & \cdots & \langle \mu_{1n}^{k},v_{1n}^{k}\rangle \\ \langle \mu_{21}^{k},v_{21}^{k}\rangle & \langle \mu_{22}^{k},v_{22}^{k}\rangle & \cdots & \langle \mu_{2n}^{k},v_{2n}^{k}\rangle \\ \vdots & \vdots & \ddots & \vdots \\ \langle \mu_{m1}^{k},v_{m1}^{k}\rangle & \langle \mu_{m2}^{k},v_{m2}^{k}\rangle & \cdots & \langle \mu_{mn}^{k},v_{mn}^{k}\rangle \end{array}\right) \end{array}$$

where $r_{ij}^{k}=\langle \mu_{ij}^{k},v_{ij}^{k}\rangle$ is an IFV representing the evaluation result of alternative *G_i_* according to attribute *A_j_*.

If the attribute weights are unknown, this MAGDM problem should be solved by steps as following.

***Step 1. Determine attribute weights***

In most cases, the weighting factor of each attribute is partly known or completely unknown due to limited time and expert knowledge. Thus, determining the weighting vector of all attributes is necessary. Several approaches have been put forward to assess the importance of all attributes in decision making.

Li et al. [62] developed the TOPSIS-based method to obtain the interval-valued weight factor for all attributes, which may cause information loss in the process of decision making. Wei [64] proposed an optical model to derive the attribute weighting vector, which was implemented by maximizing the deviation between all evaluation results under an attribute. Regarding the hesitance degree as an entropy measure, Ye [10] developed an entropy-based method to evaluate the attribute weight vector.

Note that Wei’s method [64] is based on the idea of maximizing the deviation, while Ye’s method [10] is based on the idea of minimizing the entropy. Combining Wei’s [64] and Ye’s ideas [10], Xia and Xu [9] proposed an entropy-/cross-entropy-based model to determine the attribute weighting vector, in which they utilize the cross-entropy to describe the deviation between IFVs. Borrowing the idea of Xia and Xu [9], we develop a model using the proposed distance measure *D^I^* and the knowledge measure *K^I^* to determine attribute weights.

For decision maker *E_k_*, the average divergence of alternative *G_i_* from all other alternatives under attribute *A_j_* can be measured as

(25) $$DIV_{ij}^{k}=\frac{1}{m-1}\sum_{p=1}^{m}D^{I}\left(r_{ij}^{k},r_{pj}^{k}\right)$$

Based on distance measure *D^I^* and knowledge measure *K^I^*, the average divergence and knowledge amount of all information provided by *E_k_* under attribute *A_j_* can be measured, respectively, as

(26) $$DIV_{ij}^{k}=\frac{1}{m-1}\sum_{p=1}^{m}D^{I}\left(r_{ij}^{k},r_{pj}^{k}\right)$$

(27) $$K_{j}^{k}=\sum_{p=1}^{m}K^{I}\left(r_{pj}^{k}\right)$$

Considering the weighting factor of each decision maker, we can obtain the total difference among all alternatives and the total amount of knowledge with respect to attribute *A_j_* as

(28) $$DIV_{j}=\sum_{k=1}^{s}\lambda_{k}\frac{1}{m-1}\sum_{p=1}^{m}\sum_{q=1}^{m}D^{I}\left(r_{pj}^{k},r_{qj}^{k}\right),$$

(29) $$K_{j}=\sum_{k=1}^{s}\lambda_{k}\sum_{p=1}^{m}K^{I}\left(r_{pj}^{k}\right),$$

Generally, if the evaluation information of all alternatives under an attribute is quite different from each other, it means that this attribute provides much discriminative information, and thus it should be more important. Conversely, if there is little difference among the evaluation results of all alternatives obtained with respect to one attribute, then this attribute is less important. We also have the sense that a greater amount of knowledge conveyed by the information under an attribute indicates that the information provided is more helpful for decision making. Therefore, this particular attribute is more important. Based on the above analysis, we establish an optimal model with which to calculate the weighting vector ***w*** of all attributes as

(30) $$\begin{array}{l} \max\;T=\sum_{j=1}^{n}w_{j}\sum_{k=1}^{s}\lambda_{k}\sum_{p=1}^{m}\left(K^{I}\left(r_{pj}^{k}\right)+\frac{1}{m-1}\sum_{q=1}^{m}D^{I}\left(r_{pj}^{k},r_{qj}^{k}\right)\right)\\ s.t.\;\;\;\;\left\{\begin{array}{l} \;w\in H,\;\;\;\;\\ \;\sum_{j=1}^{n}w_{j}=1,\\ w_{j}\geq 0,j=1,2,\cdots ,n. \end{array}\right. \end{array}$$

where *H* is a set that contains all of the incomplete information of an attribute weight.

In particular, if there is no additional information about the weighting vector, i.e., each attribute’s weighting factor is totally unknown, the weighting factor of attribute *A_j_* (j=1,2,⋯,n) can be calculated as

(31) $$w_{j}=\frac{\sum_{k=1}^{s}\lambda_{k}\left(K_{j}^{k}+DIV_{j}^{k}\right)}{\sum_{j=1}^{n}\sum_{k=1}^{s}\lambda_{k}\left(K_{j}^{k}+DIV_{j}^{k}\right)}=\frac{\sum_{k=1}^{s}\lambda_{k}\sum_{p=1}^{m}\left(K^{I}\left(r_{pj}^{k}\right)+\frac{1}{m-1}\sum_{q=1}^{m}D^{I}\left(r_{pj}^{k},r_{qj}^{k}\right)\right)}{\sum_{j=1}^{n}\sum_{k=1}^{s}\lambda_{k}\sum_{p=1}^{m}\left(K^{I}\left(r_{pj}^{k}\right)+\frac{1}{m-1}\sum_{q=1}^{m}D^{I}\left(r_{pj}^{k},r_{qj}^{k}\right)\right)},$$

***Step 2.*** Use the intuitionistic fuzzy weighted averaging (IFWA) operator proposed in [38] and the weighting vector $\lambda ={\left(\lambda_{1},\lambda_{2},\cdots ,\lambda_{s}\right)}^{T}$ to collect the individual intuitionistic fuzzy decision matrices $r_{ij}^{k}=\langle \mu_{ij}^{k},v_{ij}^{k}\rangle$ (k=1,2,⋯,s) into an aggregated decision matrix with intuitionistic fuzzy information, denoted *R* = (*r_ij_*)*_m__ˣn_*.

***Step 3.*** Use the aggregation operator IFWA and attribute weighting vector ***w*** to aggregate the evaluation results $r_{i1},r_{i2},\cdots ,r_{in}$ of each alternative *G_i_* (i=1,2,⋯,m) under all attributes to get an IFV *Z_i_* (i=1,2,⋯,m) denoting the aggregated evaluation result of alternative *G_i_* (i=1,2,⋯,m).

***Step 4.*** Calculate both the score function and accuracy function of IFVs $Z_{1},Z_{2},\cdots ,Z_{m}$.

***Step 5.*** Rank all alternatives according to the score function and accuracy function of IFVs $Z_{1},Z_{2},\cdots ,Z_{m}$ to obtain the priority order.

## 6. Application on Evaluation of Malicious Code Threat

Here, the method proposed in Section 5 for solving the MAGDM problems is applied on evaluation method of malicious code threat degree.

**Example** **6.** *In a battle of cyber defense, the cyber-defense unit aims to choose a target with the highest threat to attack. In cyberspace security, cyber security researchers need to evaluate the threats caused by malicious code. In the way, the most dangerous threat can be addressed first, and then the other threats can be addressed.*

The threat degrees of five malicious codes (*G*_1_, *G*_2_, *G*_3_, *G*_4_, *G*_5_) are evaluated by four experts (*E*_1_, *E*_2_, *E*_3_, *E*_4_) with respect to the following five attributes:

(1) *A*_1_, the resource consumption;
(2) *A*_2_, the destruction ability;
(3) *A*_3_, the anti-detection ability;
(4) *A*_4_, the self-starting ability;
(5) *A*_5_, the diffusion ability.

The weighting vector of four experts is $\lambda ={\left(0.3,0.2,0.3,0.2\right)}^{T}$. The associated weighting factor for the hybrid aggregation of the four experts is $\eta ={\left(0.155,\;0.345,\;0.345,\;0.155\right)}^{T}$, which is derived by the method based on normal distribution, as shown in [63]. The threat degree of each malicious code evaluated by four experts is expressed by the following four intuitionistic fuzzy decision matrices:

$$\begin{array}{l} \;\;\;\;\;\;\;\;\;\;\;\;\;\;\;\;\;\;\begin{array}{lllll} \;\;\;\;\;\;\;\;\;\;\;\;\;\;\;\;A_{1} & \;\;\;\;\;\;\;\;\;\;A_{2} & \;\;\;\;\;\;\;\;\;\;\;A_{3} & \;\;\;\;\;\;\;\;\;A_{4} & \;\;\;\;\;\;\;\;\;\;\;A_{5} \end{array}\\ R_{1}=\;\;\;\begin{array}{l} G_{1}\\ G_{2}\\ G_{3}\\ G_{4}\\ G_{5} \end{array}\;\;\;\;\left(\begin{array}{lllll} \langle 0.4,0.5\rangle & \langle 0.5,0.2\rangle & \langle 0.6,0.2\rangle & \langle 0.8,0.1\rangle & \langle 0.7,0.3\rangle \\ \langle 0.6,0.2\rangle & \langle 0.7,0.2\rangle & \langle 0.3,0.4\rangle & \langle 0.5,0.1\rangle & \langle 0.8,0.2\rangle \\ \langle 0.7,0.3\rangle & \langle 0.8,0.1\rangle & \langle 0.5,0.5\rangle & \langle 0.3,0.2\rangle & \langle 0.6,0.3\rangle \\ \langle 0.4,0.3\rangle & \langle 0.7,0.1\rangle & \langle 0.6,0.1\rangle & \langle 0.4,0.3\rangle & \langle 0.9,0.1\rangle \\ \langle 0.8,0.1\rangle & \langle 0.3,0.4\rangle & \langle 0.4,0.5\rangle & \langle 0.7,0.2\rangle & \langle 0.5,0.2\rangle \end{array}\right) \end{array},\;\begin{array}{l} \;\;\;\;\;\;\;\;\;\;\;\;\;\;\;\;\;\;\begin{array}{lllll} \;\;\;\;\;\;\;\;\;\;\;\;\;\;\;\;A_{1} & \;\;\;\;\;\;\;\;\;\;A_{2} & \;\;\;\;\;\;\;\;\;\;\;A_{3} & \;\;\;\;\;\;\;\;\;A_{4} & \;\;\;\;\;\;\;\;\;\;\;A_{5} \end{array}\\ R_{2}=\;\;\;\begin{array}{l} G_{1}\\ G_{2}\\ G_{3}\\ G_{4}\\ G_{5} \end{array}\;\;\;\;\left(\begin{array}{lllll} \langle 0.5,0.3\rangle & \langle 0.6,0.1\rangle & \langle 0.7,0.3\rangle & \langle 0.7,0.1\rangle & \langle 0.8,0.2\rangle \\ \langle 0.7,0.2\rangle & \langle 0.6,0.2\rangle & \langle 0.4,0.4\rangle & \langle 0.6,0.2\rangle & \langle 0.7,0.3\rangle \\ \langle 0.5,0.3\rangle & \langle 0.7,0.2\rangle & \langle 0.6,0.3\rangle & \langle 0.4,0.2\rangle & \langle 0.6,0.1\rangle \\ \langle 0.5,0.4\rangle & \langle 0.8,0.1\rangle & \langle 0.4,0.2\rangle & \langle 0.7,0.2\rangle & \langle 0.7,0.3\rangle \\ \langle 0.7,0.3\rangle & \langle 0.5,0.4\rangle & \langle 0.6,0.3\rangle & \langle 0.6,0.2\rangle & \langle 0.5,0.1\rangle \end{array}\right) \end{array},\;\begin{array}{l} \;\;\;\;\;\;\;\;\;\;\;\;\;\;\;\;\;\;\begin{array}{lllll} \;\;\;\;\;\;\;\;\;\;\;\;\;\;\;\;A_{1} & \;\;\;\;\;\;\;\;\;\;A_{2} & \;\;\;\;\;\;\;\;\;\;\;A_{3} & \;\;\;\;\;\;\;\;\;A_{4} & \;\;\;\;\;\;\;\;\;\;\;A_{5} \end{array}\\ R_{3}=\;\;\;\begin{array}{l} G_{1}\\ G_{2}\\ G_{3}\\ G_{4}\\ G_{5} \end{array}\;\;\;\;\left(\begin{array}{lllll} \langle 0.6,0.3\rangle & \langle 0.5,0.2\rangle & \langle 0.6,0.4\rangle & \langle 0.8,0.1\rangle & \langle 0.7,0.3\rangle \\ \langle 0.8,0.2\rangle & \langle 0.5,0.3\rangle & \langle 0.6,0.4\rangle & \langle 0.5,0.2\rangle & \langle 0.6,0.3\rangle \\ \langle 0.6,0.1\rangle & \langle 0.8,0.2\rangle & \langle 0.7,0.3\rangle & \langle 0.4,0.2\rangle & \langle 0.8,0.1\rangle \\ \langle 0.6,0.3\rangle & \langle 0.6,0.1\rangle & \langle 0.5,0.4\rangle & \langle 0.9,0.1\rangle & \langle 0.5,0.2\rangle \\ \langle 0.8,0.1\rangle & \langle 0.6,0.2\rangle & \langle 0.7,0.3\rangle & \langle 0.5,0.2\rangle & \langle 0.7,0.1\rangle \end{array}\right) \end{array},\;\begin{array}{l} \;\;\;\;\;\;\;\;\;\;\;\;\;\;\;\;\;\;\begin{array}{lllll} \;\;\;\;\;\;\;\;\;\;\;\;\;\;\;\;A_{1} & \;\;\;\;\;\;\;\;\;\;A_{2} & \;\;\;\;\;\;\;\;\;\;\;A_{3} & \;\;\;\;\;\;\;\;\;A_{4} & \;\;\;\;\;\;\;\;\;\;\;A_{5} \end{array}\\ R_{4}=\;\;\;\begin{array}{l} G_{1}\\ G_{2}\\ G_{3}\\ G_{4}\\ G_{5} \end{array}\;\;\;\;\left(\begin{array}{lllll} \langle 0.3,0.4\rangle & \langle 0.9,0.1\rangle & \langle 0.8,0.1\rangle & \langle 0.5,0.5\rangle & \langle 0.4,0.6\rangle \\ \langle 0.7,0.1\rangle & \langle 0.7,0.3\rangle & \langle 0.4,0.2\rangle & \langle 0.8,0.2\rangle & \langle 0.3,0.1\rangle \\ \langle 0.4,0.1\rangle & \langle 0.5,0.2\rangle & \langle 0.8,0.1\rangle & \langle 0.6,0.2\rangle & \langle 0.6,0.3\rangle \\ \langle 0.8,0.2\rangle & \langle 0.5,0.1\rangle & \langle 0.6,0.4\rangle & \langle 0.7,0.2\rangle & \langle 0.7,0.2\rangle \\ \langle 0.6,0.1\rangle & \langle 0.8,0.2\rangle & \langle 0.7,0.2\rangle & \langle 0.6,0.3\rangle & \langle 0.8,0.1\rangle \end{array}\right) \end{array}$$

**Case** **1.** *First, we suppose that the weight of each attribute is totally unknown.*

We then use the proposed method shown in Equation (31) to establish the weighting vectors of five attributes. We solve this problem according to the next steps:

(1) Using the distance measure *D^I^* and knowledge measure *K^I^* to get the average divergence and the amount of knowledge under all attributes for all decision makers, we obtain the divergence and knowledge matrix, respectively, as
  $$DIV=\;\;\left(\begin{array}{lllll} 1.0067 & 0.9763 & 0.9274 & 0.9013 & 0.7288\\ 0.5312 & 0.7012 & 0.5487 & 0.4928 & 0.4707\\ 0.6048 & 0.5614 & 0.8524 & 0.9013 & 0.6275\\ 0.9661 & 0.7473 & 0.8370 & 0.7270 & 1.1840 \end{array}\right),\;K=\;\;\left(\begin{array}{lllll} 2.7112 & 2.8317 & 2.4047 & 2.4629 & 3.1393\\ 2.5628 & 2.9237 & 2.3939 & 2.6850 & 2.9527\\ 3.0721 & 2.6788 & 2.8276 & 2.4629 & 2.9745\\ 2.6547 & 3.0779 & 3.0100 & 2.8944 & 2.6928 \end{array}\right),$$

The elements *div_ij_* in matrix *DIV* represents the whole average divergence provided by *D_i_* under *A_j_*, and *k_ij_* in matrix *K* represents the knowledge amount provided by *D_i_* under *A_j_*.

(2) Given the weight vector $\lambda ={\left(0.3,0.2,0.3,0.2\right)}^{T}$, we obtain the attribute weight vector based on Equation (31):
  $$w={\left(0.2011,0.2036,0.1955,0.1908,0.2090\right)}^{T}.$$
(3) Collecting all decision makers’ decision matrices based on the proposed IFWA operator, we can get the aggregated decision matrix as:
  $$\begin{array}{l} \;\;\;\;\;\;\;\;\;\;\;\;\;\;\;\;\;\;\;\;\;\;\;\;\;\;\begin{array}{lllll} \;\;\;\;\;\;\;\;\;\;\;\;\;A_{1} & \;\;\;\;\;\;\;\;\;\;\;\;\;\;\;\;\;\;\;\;\;\;A_{2} & \;\;\;\;\;\;\;\;\;\;\;\;\;\;\;\;\;\;\;\;\;\;\;A_{3} & \;\;\;\;\;\;\;\;\;\;\;\;\;\;\;\;\;\;\;\;\;\;A_{4} & \;\;\;\;\;\;\;\;\;\;\;\;\;\;\;\;\;\;\;\;\;\;A_{5} \end{array}\\ R=\;\;\;\begin{array}{l} G_{1}\\ G_{2}\\ G_{3}\\ G_{4}\\ G_{5} \end{array}\;\;\;\;\left(\begin{array}{lllll} \langle 0.4717,0.3704\rangle & \langle 0.6534,0.1516\rangle & \langle 0.7330,0.1534\rangle & \langle 0.7395,0.1380\rangle & \langle 0.6822,0.3178\rangle \\ \langle 0.7104,0.1741\rangle & \langle 0.6296,0.2449\rangle & \langle 0.4050,0.2828\rangle & \langle 0.6019,0.1320\rangle & \langle 0.6569,0.2132\rangle \\ \langle 0.5839,0.1732\rangle & \langle 0.7395,0.1625\rangle & \langle 0.5795,0.2486\rangle & \langle 0.3931,0.2000\rangle & \langle 0.6751,0.1732\rangle \\ \langle 0.5888,0.2930\rangle & \langle 0.6660,0.1000\rangle & \langle 0.7138,0.1516\rangle & \langle 0.5453,0.2551\rangle & \langle 0.7485,0.1762\rangle \\ \langle 0.7509,0.1246\rangle & \langle 0.5963,0.2828\rangle & \langle 0.5440,0.2855\rangle & \langle 0.6634,0.2169\rangle & \langle 0.6429,0.1231\rangle \end{array}\right) \end{array}.$$
(4) Based on the vector $w={\left(0.2011,0.2036,0.1955,0.1908,0.2090\right)}^{T}$, we aggregate the threat degree of each target under all attributes using the IFWA operator to obtain
  $$Z_{1}=\langle 0.6666,0.2085\rangle ,\;Z_{2}=\langle 0.6141,0.2031\rangle ,\;Z_{3}=\langle 0.6132,0.1886\rangle ,\;Z_{4}=\langle 0.6622,0.1812\rangle ,\;Z_{5}=\langle 0.6421,0.1920\rangle .$$
(5) The score function of *Z*_1_, *Z*_2_, *Z*_3_, *Z*_4_, *Z*_5_ can be calculated as:
  $$S(Z_{1})=0.4581,\;S(Z_{2})=0.4111,\;S(Z_{3})=0.4246,\;S(Z_{4})=0.4810,\;S(Z_{5})=0.4501.$$
(6) According to the score grades, we obtain the ranking order *R* of all malicious codes’ threat degree as
  $$G_{4}≻G_{1}≻G_{5}≻G_{3}≻G_{2}.$$

Based on the method proposed in [9], when $E_{M}^{1.5}$ and $CE_{M}^{1.5}$ are used, the attribute weights are obtained as $w_{a}={\left(0.1940,0.2238,0.1330,0.2117,0.2375\right)}^{T}$, and the final ranking order is *R_a_*: $G_{4}≻G_{5}≻G_{1}≻G_{3}≻G_{2}$. When $E_{N}^{1}$ and $CE_{N}^{1}$ are used, the attribute weights are obtained as $w_{b}={\left(0.1931,0.2219,0.1325,0.2133,0.2392\right)}^{T}$, and the final ranking order is *R_b_*: $G_{4}≻G_{5}≻G_{1}≻G_{3}≻G_{2}$. It is notable that the final ranking order obtained using the method proposed in Section 5 is not completely identical to that obtained in [9]. However, all methods can be used to obtain the same optimal alternative, *G*_4_. Since the solving the MAGDM problem is aimed at obtaining the best choice, the order of other alternatives may not be of concern. We can use the similarity between two weighting vectors, which is defined as the cosine value of the angle between them, denoted *Sim*:

(32) $$Sim(w_{1},w_{2})=\frac{w_{1}^{T}w_{2}}{\sqrt{\left(w_{1}^{T}w_{1}\right)\left(w_{1}^{T}w_{2}\right)}},$$

The consensus level between two ranking orders *R*_1_ and *R*_2_ is calculated by Spearman’s rank correlation coefficient [65]:

(33) $$\rho (R_{1},R_{2})=1-6\sum_{i=1}^{p}{\left(r_{i}^{\left(1\right)}-r_{i}^{\left(2\right)}\right)}^{2}/p(p^{2}-1),$$

where *p* is the number of alternatives; $r_{i}^{\left(1\right)}$ and $r_{i}^{\left(2\right)}$ are the positions of alternative *G_i_* in respective ranking order *R*_1_ and *R*_2_.

We then obtain

$$Sim(w,w_{a})=0.9863;Sim(w,w_{b})=0.9859;\rho (R,R_{a})=\rho (R,R_{b})=0.9.$$

These results indicate that the attribute weights obtained by the proposed method are quite similar to those yielded in [9]. Moreover, the ranking orders are at a high consensus level. It is demonstrated that the proposed method is effective for solving MAGDM problems.

**Case** **2.** *We suppose that the attribute weights are partially known by some relations as following:*

$$H=\{w_{1}\geq 0.1;0.2\leq w_{2}\leq 0.3;w_{3}\geq 0.15;0.2\leq w_{4}\leq 0.3;0.3\leq w_{5}\leq 0.4\}.$$

We can then use the following optimal model to get the attribute weighting vector:

$$\begin{array}{l} \max\;\;\;T=(3.5614,3.6045,3.4614,3.3783,3.7011)w\\ s.t.\;\;\;\;\left\{\begin{array}{l} \;w\in H,\;\;\;\;\\ \;\sum_{j=1}^{5}w_{j}=1,\\ w_{j}\geq 0,j=1,2,\cdots ,5. \end{array}\right. \end{array},$$

and we obtain the weighting vector as $w={\left(0.1,0.2,0.15,0.2,0.35\right)}^{T}$.

Using the weighting vector ***w***, we obtain the aggregated threat grades of each malicious code by the IFWA operator:

$$Z_{1}=\langle 0.6815,0.2110\rangle ,\;Z_{2}=\langle 0.6168,0.2036\rangle ,\;Z_{3}=\langle 0.6247,0.1858\rangle ,\;Z_{4}=\langle 0.6791,0.1743\rangle ,\;Z_{5}=\langle 0.6334,0.1849\rangle ,$$

and their scores are calculated as

$$S(Z_{1})=0.4704,\;S(Z_{2})=0.4131,\;S(Z_{3})=0.4389,\;S(Z_{4})=0.5048,\;S(Z_{5})=0.4484,$$

respectively. Then, we obtain the ranking order as

$$G_{4}≻G_{1}≻G_{5}≻G_{3}≻G_{2}.$$

If there is only one expert in MAGDM problems, we do not need to fuse the results of different experts. Thus, we can deal with such cases by evaluating attribute weight vector and then aggregating all the results under different attributes. We will use another example to compare the proposed methods with other methods.

**Example** **7.** *The cyber-defense unit will attack the malicious code with the maximum threat grade. In cyberspace security, cyber security researchers evaluate their own protection capabilities by evaluating malicious codes, and can judge the order in which malicious codes are difficult to be discovered in the system.*

There are pieces of five malicious code for their choice. The following five types of malicious code include:

Five malicious code are presented as:

*G*_1_, a backdoor;
*G*_2_, a Trojan-PWS;
*G*_3_, a Worm;
*G*_4_, a Trojan-Spy;
*G*_5_, a Trojan-Downloader.

The cyber security researchers evaluates these five malicious code based on four attributes, which are the following:

*A*_1_, the resource consumption;
*A*_2_, the self-starting ability;
*A*_3_, the con-cealment ability;
*A*_4_, the self-protection ability.

The results of evaluation using intuitionistic fuzzy information are

$$\begin{array}{l} \;\;\;\;\;\;\;\;\;\;\;\;\;\;\;\;\;\;\;\begin{array}{llll} \;\;\;\;\;\;\;\;\;\;\;\;A_{1} & \;\;\;\;\;\;\;\;\;\;\;\;A_{2} & \;\;\;\;\;\;\;\;\;\;A_{3} & \;\;\;\;\;\;\;\;A_{4} \end{array}\\ R=\;\;\;\begin{array}{l} G_{1}\\ G_{2}\\ G_{3}\\ G_{4}\\ G_{5} \end{array}\;\;\;\;\left(\begin{array}{llll} \langle 0.5,0.4\rangle & \langle 0.6,0.3\rangle & \langle 0.3,0.6\rangle & \langle 0.2,0.7\rangle \\ \langle 0.7,0.3\rangle & \langle 0.7,0.2\rangle & \langle 0.7,0.2\rangle & \langle 0.4,0.5\rangle \\ \langle 0.6,0.4\rangle & \langle 0.5,0.4\rangle & \langle 0.5,0.3\rangle & \langle 0.6,0.3\rangle \\ \langle 0.8,0.1\rangle & \langle 0.6,0.3\rangle & \langle 0.3,0.4\rangle & \langle 0.2,0.6\rangle \\ \langle 0.6,0.2\rangle & \langle 0.4,0.3\rangle & \langle 0.7,0.1\rangle & \langle 0.5,0.3\rangle \end{array}\right) \end{array}.$$

**Case** **1.** *There is no information available for all attributes’ weights.*

(1) Using the distance measure *D^I^* and knowledge measure *K^I^* to get the average divergence and the amount of knowledge under all attributes, we obtain the divergence and knowledge matrix, respectively, shown as
  $$DIV=\;\;\left(0.7468,0.5484,1.2190,1.1117\right),\;K=\left(2.8798,2.4825,2.5963,2.5674\right)$$

The elements *div_i_* and *k_i_* in vector *DIV* and *K* represent the average divergence degree and knowledge quantity under attribute *A_i_*, respectively.

(2) The weight factor of attribute *A_i_* can be calculated as
  $$w_{i}=\frac{div_{i}+k_{i}}{\sum_{i=1}^{n}\left(div_{i}+k_{i}\right)},\;i=1,2,\cdots ,5$$

We then obtain the weighting vector as ***w*** = (0.2563,0.2142,0.2696,0.2600)*^T^*.

(3) Aggregate the evaluation results of each target under all attributes based on the weighting vector ***w*** and the IFWA operator. The final threat grades of five malicious code are:
  $$Z_{1}=\langle 0.4102,0.4852\rangle ,\;Z_{2}=\langle 0.6408,0.2816\rangle ,\;Z_{3}=\langle 0.5544,0.3435\rangle ,\;Z_{4}=\langle 0.5337,0.2930\rangle ,\;Z_{5}=\langle 0.5722,0.2011\rangle .$$
(4) The score grades of all alternatives are computed as
  $$S(Z_{1})=-0.0750,\;S(Z_{2})=0.3592,\;S(Z_{3})=0.2109,\;S(Z_{4})=0.2407,\;S(Z_{5})=0.3711.$$
(5) Thus, we rank all alternatives in order *R* as $G_{5}≻G_{2}≻G_{4}≻G_{3}≻G_{1}$.

For further analysis, we compare these results with the solutions for Xia and Xu’s method [9]. The weighting vector that they obtained is $w_{c}={\left(0.2659,0.2486,0.2370,0.2486\right)}^{T}$ and the ranking order is *R_c_*: $G_{5}≻G_{2}≻G_{3}≻G_{4}≻G_{1}$. We note that these ranking orders are slightly diverse due to the distinction between intuitionistic fuzzy measures used, but they obtain the same optimal alternative *G*_5_.

We also obtain $Sim(w,w_{c})=0.9975$ and $\rho (R,R_{c})=0.9$, indicating that the results achieved based on the method proposed in Section 5 are quite close to the results in [9].

**Case** **2.** *Suppose that partially information on the attribute is available as:*

$$H=\{0.15\leq w_{1}\leq 0.2;0.16\leq w_{2}\leq 0.18;0.3\leq w_{3}\leq 0.35;0.3\leq w_{4}\leq 0.45\},$$

*we then can build an optimal model to calculate the attribute weight:*

$$\begin{array}{l} \max\;\;\;T=(3.6266,3.0310,3.8153,3.6791)w\\ s.t.\;\;\;\;\left\{\begin{array}{l} \;w\in H,\;\;\;\;\\ \;\sum_{j=1}^{4}w_{j}=1. \end{array}\right. \end{array}$$

*and can obtain the weight vector:*

$$w={\left(0.20,0.16,0.34,0.30\right)}^{T}.$$

Aggregating the threat grades of each target under all attributes using the IFWA operator, we obtain

$$Z_{1}=\langle 0.3371,0.5186\rangle ,\;Z_{2}=\langle 0.6307,0.2855\rangle ,\;Z_{3}=\langle 0.5528,0.3327\rangle ,\;Z_{4}=\langle 0.4814,0.3270\rangle ,\;Z_{5}=\langle 0.5862,0.1904\rangle .$$

The score grades of these IFVs representing each target’s threat degree can be obtained as

$$S(Z_{1})=-0.1415,\;S(Z_{2})=0.3451,\;S(Z_{3})=0.2201,\;S(Z_{4})=0.1545,\;S(Z_{5})=0.3958.$$

By comparing the score grades of five IFVs, the ranking order of these five malicious codes’ threat degree can be obtained as: $G_{5}≻G_{2}≻G_{3}≻G_{4}≻G_{1}$.

Using the method proposed in [9], the attribute weights can be yielded as $w_{d}={\left(0.19,0.16,0.35,0.30\right)}^{T}$, and the corresponding ranking order is *R_d_*: $G_{5}≻G_{2}≻G_{3}≻G_{4}≻G_{1}$.

It is shown that the weighting vector obtained by the proposed method is much close to that obtained by Xia and Xu in [9] when partial information on the attribute weight is provided. We calculate the similarity degree between them as $Sim(w,w_{c})=0.9996$. It can also be seen that the order yielded by our proposed method is identical to *R_d_*, a phenomenon that appears to be caused by the incomplete information.

These illustrative examples reveal the necessity of utilizing distance and knowledge measures to establish the attribute weights. They further demonstrate that our method proposed here reasonably and effectively handles intuitionistic fuzzy MAGDM problems. The applicability of our proposed knowledge measure is also illustrated. In the method proposed in [9], we note that they used more complex entropy/cross-entropy measures with additional parameters, but without specific physical meaning. Moreover, the hybrid aggregation operator used in [8] needs an associated weight vector to aggregate intuitionistic fuzzy information. Compared with these entropy/cross-entropy measures in [9], our developed distance and knowledge measures with relatively concise simple expressions and specific physical meaning can also obtain reasonable solutions with the help of the original IFWA operator. Thus, our proposed method seems to be more practical and easier to implement to solve MAGDM problems.

## 7. Conclusions

In this paper, we propose a knowledge measure based on our proposed intuitionistic fuzzy distance measure for the purpose of measuring the knowledge amount of AIFSs more accurately. The axiomatic definition of knowledge measure is refined from a more general view, after which we investigate the properties of the new distance-based knowledge measure. Mathematical analysis and numerical examples are provided to illustrate the proposed knowledge measure’s properties. To demonstrate the applicability of the proposed distance-based knowledge measure, we apply it to develop a new method of solving MAGDM problems with intuitionistic fuzzy information. Application examples combined with comparative analysis illustrate the effectiveness and rationality of our method.

We only present a knowledge measure based on our proposed distance measure in this paper. The main feature of the proposed knowledge measure lies in its succinct expression, good properties, and evident physical significance. This is a new perspective to considering knowledge measure and uncertainty measure. There must be other kinds of knowledge measures used if other distance measures are applied. Exploration on the reasonable distance measure is critical for the definition of knowledge measure. Conversely, based on the relation on distance measure and uncertainty measure, we can also develop new distance measure based on some reasonable knowledge measures. Furthermore, syncretic research on distance measure, similarity measure, knowledge measure, and uncertainty measure is also attractive and worthy.

## Figures and Tables

**Table 1:** Entropy/knowledge measures used for comparative analysis.

Authors	Entropy/Knowledge Measure
Zeng and Li [56]	$E_{ZL}(A)=1-\frac{1}{n}\sum_{i=1}^{n}\left|\mu_{A}(x_{i})-v_{A}(x_{i})\right|$
Zhang, Zhang, and Mei [57]	$E_{ZA}(A)=1-\sqrt{\frac{2}{n}\sum_{i=1}^{n}{\left|\mu_{A}(x_{i})-0.5\right|}^{2}+{\left|1-v_{A}(x_{i})-0.5\right|}^{2}}$
Zhang, Zhang, and Mei [57]	$E_{ZB}(A)=1-\frac{1}{n}\sum_{i=1}^{n}\left(\left|\mu_{A}(x_{i})-0.5\right|+\left|1-v_{A}(x_{i})-0.5\right|\right)$
Zhang, Zhang, and Mei [57]	$E_{ZC}(A)=1-\frac{2}{n}\sum_{i=1}^{n}\max\left(\left|\mu_{A}(x_{i})-0.5\right|,\left|1-v_{A}(x_{i})-0.5\right|\right)$
Zhang, Zhang, and Mei [57]	$E_{ZD}(A)=1-\sqrt{\frac{4}{n}\sum_{i=1}^{n}\max\left({\left|\mu_{A}(x_{i})-0.5\right|}^{2},{\left|1-v_{A}(x_{i})-0.5\right|}^{2}\right)}$
Zhang, Zhang, and Mei [57]	$E_{ZE}(A)=1-\frac{2}{n}\sum_{i=1}^{n}\left(\frac{\left|\mu_{A}(x_{i})-0.5\right|+\left|1-v_{A}(x_{i})-0.5\right|}{4}+\frac{\max\left(\left|\mu_{A}(x_{i})-0.5\right|,\left|1-v_{A}(x_{i})-0.5\right|\right)}{2}\right)$
Burillo and Bustince [21]	$E_{BB}(A)=\frac{1}{n}\sum_{i=1}^{n}\left(1-\mu_{A}(x_{i})-v_{A}(x_{i})\right)$
Szmidt and Kacprzyk [22]	$E_{SK}(A)=\frac{1}{n}\sum_{i=1}^{n}\frac{\min\left(\mu_{A}(x_{i}),v_{A}(x_{i})\right)+\pi_{A}(x_{i})}{\max\left(\mu_{A}(x_{i}),v_{A}(x_{i})\right)+\pi_{A}(x_{i})}$
Hung and Yang [60]	$E_{HC}^{2}(A)=\frac{1}{n}\sum_{i=1}^{n}\left(1-{\left(\mu_{A}(x_{i})\right)}^{2}-{\left(v_{A}(x_{i})\right)}^{2}-{\left(\pi_{A}(x_{i})\right)}^{2}\right)$
Hung and Yang [60]	$E_{S}(A)=-\frac{1}{n}\sum_{i=1}^{n}\left(\mu_{A}(x_{i})\ln\mu_{A}(x_{i})+v_{A}(x_{i})\ln v_{A}(x_{i})+\pi_{A}(x_{i})\ln\pi_{A}(x_{i})\right)$
Vlachos and Sergiadis [61]	$E_{VS}(A)=-\frac{1}{n\ln2}\sum_{i=1}^{n}\left(\mu_{A}(x_{i})\ln\mu_{A}(x_{i})+v_{A}(x_{i})\ln v_{A}(x_{i})+\left(1-\pi_{A}(x_{i})\right)\ln\left(1-\pi_{A}(x_{i})\right)\right)+\frac{1}{n}\sum_{i=1}^{n}\pi_{A}(x_{i})$
Zhang and Jiang [58]	$E_{ZJ}(A)=\frac{1}{n}\sum_{i=1}^{n}\frac{\min\left(\mu_{A}(x_{i}),v_{A}(x_{i})\right)}{\max\left(\mu_{A}(x_{i}),v_{A}(x_{i})\right)}$
Li, Deng, Li, et al. [55]	$E_{LDL}(A)=1-\frac{1}{2n}\sum_{i=1}^{n}\left({\left|\mu_{A}(x_{i})-v_{A}(x_{i})\right|}^{3}+\left|\mu_{A}(x_{i})-v_{A}(x_{i})\right|\right)$
Szmidt, Kacprzyk, andBujnowski [27]	$K_{SKB}(A)=1-\frac{1}{2n}\left(\sum_{i=1}^{n}\frac{\min\left(\mu_{A}(x_{i}),v_{A}(x_{i})\right)+\pi_{A}(x_{i})}{\max\left(\mu_{A}(x_{i}),v_{A}(x_{i})\right)+\pi_{A}(x_{i})}+\pi_{A}(x_{i})\right)$
Nguyen [30]	$K_{N}(A)=\frac{1}{n\sqrt{2}}\sum_{i=1}^{n}\sqrt{{\left(\mu_{A}(x_{i})\right)}^{2}+{\left(v_{A}(x_{i})\right)}^{2}+{\left(\mu_{A}(x_{i})+v_{A}(x_{i})\right)}^{2}}$
Guo [31]	$K_{G}(A)=1-\frac{1}{2n}\sum_{i=1}^{n}\left(1-\left|\mu_{A}(x_{i})-v_{A}(x_{i})\right|\right)\left(1+\pi_{A}(x_{i})\right)$

**Table 2:** Comparative results of all AIFSs with respect to *A* (counter-intuitive results are in bold type).

	*A* ^0.5^	*A*	*A* ^2^	*A* ^3^	*A* ^4^
*E_ZL_*	**0.4156**	**0.4200**	0.2380	0.1546	0.1217
*E_ZA_*	0.3214	0.3043	0.1974	0.1330	0.0979
*E_ZB_*	**0.4156**	**0.4200**	0.2380	0.1546	0.1217
*E_ZC_*	0.3338	0.3200	0.1400	0.0612	0.0283
*E_ZD_*	0.2777	0.2463	0.1188	0.0562	0.0271
*E_ZE_*	0.3747	0.3700	0.1890	0.1079	0.0750
*E_BB_*	**0.0818**	**0.1000**	0.0980	0.0934	0.0934
*E_SK_*	**0.3446**	**0.3740**	0.1970	0.1309	0.1094
*E_HC_*	**0.3416**	**0.3440**	0.2610	0.1993	0.1613
*E_S_*	**0.5811**	**0.5874**	0.4555	0.3489	0.2778
*E_VS_*	0.5518	0.5217	0.3491	0.2357	0.1733
*E_ZJ_*	**0.2851**	**0.3050**	0.1042	0.0383	0.0161
*E_LDL_*	0.5083	0.5019	0.3454	0.2516	0.2001
*K_SKB_*	**0.7868**	**0.7630**	0.8525	0.8879	0.8986
*K_N_*	**0.8585**	**0.8471**	0.8738	0.8927	0.8999
*K_G_*	**0.7665**	**0.7610**	0.8651	0.9108	0.9257
*K_I_*	0.7059	0.7098	0.8066	0.8624	0.8858

**Table 3:** Comparative results of all AIFSs with respect to B (counter-intuitive results are in bold type).

	*B* ^0.5^	*B*	*B* ^2^	*B* ^3^	*B* ^4^
*E_ZL_*	**0.4291**	**0.4400**	0.2160	0.1364	0.1082
*E_ZA_*	0.3310	0.3072	0.1868	0.1193	0.0859
*E_ZB_*	**0.4291**	**0.4400**	0.2160	0.1364	0.1082
*E_ZC_*	0.3608	0.3600	0.1400	0.0612	0.0283
*E_ZD_*	0.2960	0.2517	0.1188	0.0562	0.0271
*E_ZE_*	**0.3950**	**0.4000**	0.1780	0.0988	0.0683
*E_BB_*	**0.0683**	**0.0800**	0.0760	0.0752	0.0800
*E_SK_*	**0.3518**	**0.4073**	0.1677	0.1101	0.0950
*E_HC_*	0.3355	0.3280	0.2328	0.1708	0.1379
*E_S_*	0.5494	0.5374	0.3929	0.2905	0.2295
*E_VS_*	0.5640	0.5233	0.3369	0.2212	0.1612
*E_ZJ_*	**0.3042**	**0.3450**	0.0927	0.0349	0.0151
*E_LDL_*	0.5191	0.5120	0.3279	0.2290	0.1791
*K_SKB_*	**0.7899**	**0.7563**	0.8782	0.9074	0.9125
*K_N_*	**0.8680**	**0.8641**	0.8950	0.9108	0.9133
*K_G_*	**0.7633**	**0.7600**	0.8828	0.9230	0.9337
*K_I_*	0.7038	0.7182	0.8272	0.8804	0.8992

## Data Availability

The data used to support the findings of this study are available from the corresponding author upon request.

## Appendix A

**Proof** **of** **Theorem** **1.** (1) Given $D^{I}(A,B)=0$, we obtain $\frac{\mu_{A}(x_{i})-v_{A}(x_{i})}{2}-\frac{\mu_{B}(x_{i})-v_{B}(x_{i})}{2}=0$ and $\frac{\mu_{A}(x_{i})+v_{A}(x_{i})}{2}-\frac{\mu_{B}(x_{i})+v_{B}(x_{i})}{2}=0$ ∀i∈{1,2,⋯,n}, which can be written identically as

$$\mu_{A}(x_{i})-v_{A}(x_{i})=\mu_{B}(x_{i})-v_{B}(x_{i}),\;\mu_{A}(x_{i})+v_{A}(x_{i})=\mu_{B}(x_{i})+v_{B}(x_{i}).$$

We then obtain $\mu_{A}(x_{i})=\mu_{B}(x_{i})$ and $v_{A}(x_{i})=v_{B}(x_{i})$ by adding and subtracting the above equations, respectively, i=1,2,⋯,n. Hence, for all elements x∈X, $\mu_{A}(x)=\mu_{B}(x)$ and $v_{A}(x)=v_{B}(x)$ hold simultaneously, which indicates that *A*=*B*. For two AIFSs, *A* and *B*, defined in $X=\{x_{1},x_{2},\cdots ,x_{n}\}$, we have the following relation:

$$A=B\Rightarrow \forall i\in \{1,2,\cdots ,n\},\mu_{A}(x_{i})=\mu_{B}(x_{i}),v_{A}(x_{i})=v_{B}(x_{i})\Rightarrow D^{I}(A,B)=0.$$

We can conclude from the above analysis that $D^{I}(A,B)=0\Leftrightarrow A=B$. (2) It is straightforward that $D^{I}(A,B)=D^{I}(B,A)$. (3) Three AIFSs, A, B, and C defined in $X=\{x_{1},x_{2},\cdots ,x_{n}\}$, can be expressed as $A=\left\{\langle x,\mu_{A}(x),v_{A}(x)\rangle \left|x\in X\right.\right\}$, $B=\left\{\langle x,\mu_{B}(x),v_{B}(x)\rangle \left|x\in X\right.\right\}$, and $C=\left\{\langle x,\mu_{C}(x),v_{C}(x)\rangle \left|x\in X\right.\right\}$, respectively. Considering the condition A⊆B⊆C, we have the relations $\mu_{A}(x_{i})\leq \mu_{B}(x_{i})\leq \mu_{C}(x_{i})$ and $v_{A}(x_{i})\geq v_{B}(x_{i})\geq v_{C}(x_{i})$. The distance between AIFSs *A* and *B* can be written as $\begin{array}{l} D^{I}(A,B)=\\ \frac{1}{n}\sum_{i=1}^{n}\sqrt{{\left(\frac{\mu_{A}(x_{i})-v_{A}(x_{i})}{2}-\frac{\mu_{B}(x_{i})-v_{B}(x_{i})}{2}\right)}^{2}+\frac{1}{3}{\left(\frac{\mu_{A}(x_{i})+v_{A}(x_{i})}{2}-\frac{\mu_{B}(x_{i})+v_{B}(x_{i})}{2}\right)}^{2}} \end{array}$. The distance between AIFSs *A* and *C* can be written as $\begin{array}{l} D^{I}(A,C)=\\ \frac{1}{n}\sum_{i=1}^{n}\sqrt{{\left(\frac{\mu_{A}(x_{i})-v_{A}(x_{i})}{2}-\frac{\mu_{C}(x_{i})-v_{C}(x_{i})}{2}\right)}^{2}+\frac{1}{3}{\left(\frac{\mu_{A}(x_{i})+v_{A}(x_{i})}{2}-\frac{\mu_{C}(x_{i})+v_{C}(x_{i})}{2}\right)}^{2}} \end{array}$. The distance between AIFSs *B* and *C* can be written as $\begin{array}{l} D^{I}(B,C)=\\ \frac{1}{n}\sum_{i=1}^{n}\sqrt{{\left(\frac{\mu_{B}(x_{i})-v_{B}(x_{i})}{2}-\frac{\mu_{C}(x_{i})-v_{C}(x_{i})}{2}\right)}^{2}+\frac{1}{3}{\left(\frac{\mu_{B}(x_{i})+v_{B}(x_{i})}{2}-\frac{\mu_{C}(x_{i})+v_{C}(x_{i})}{2}\right)}^{2}} \end{array}$. We then construct a function f(x,y) with two variables as

$$f(x,y)={\left(\left(x-y)-(a-b\right)\right)}^{2}+\frac{1}{3}{\left(\left(x+y)-(a+b\right)\right)}^{2},$$

where 0≤x≤1, 0≤y≤1, 0≤a≤1, and 0≤b≤1. The partial derivatives for variables *x* and *y* can be obtained as follows:

$$\begin{array}{l} \frac{\partial f}{\partial x}=2\left(\left(x-y)-(a-b\right)\right)+\frac{2}{3}\left(\left(x+y)-(a+b\right)\right)\\ \;\;\;\;\;=\frac{8}{3}\left(x-a\right)+\frac{4}{3}\left(b-y\right)\\ \;\;\;\;\;=\frac{4}{3}\left(2\left(x-a\right)+\left(b-y\right)\right) \end{array},\;\begin{array}{l} \frac{\partial f}{\partial y}=-2\left(\left(x-y)-(a-b\right)\right)+\frac{2}{3}\left(\left(x+y)-(a+b\right)\right)\\ \;\;\;\;\;=\frac{8}{3}\left(y-b\right)+\frac{4}{3}\left(a-x\right)\\ \;\;\;\;\;=\frac{4}{3}\left(2\left(y-b\right)+\left(a-x\right)\right) \end{array}.$$

(i) Given the conditions 0≤a≤x≤1 and 0≤y≤b≤1, we obtain that ∂f/∂x≥0 and ∂f/∂y≤0. Thus, f(x,y) is an increasing function of variable *x* and a decreasing function of variable *y*. Letting $a=\mu_{A}(x_{i})$ and $b=v_{A}(x_{i})$ then $a=\mu_{A}(x_{i})\leq \mu_{B}(x_{i})\leq \mu_{C}(x_{i})$ and $b=v_{A}(x_{i})\geq v_{B}(x_{i})\geq v_{C}(x_{i})$, $i\in \left\{1,2,\cdots ,n\right\}$. Considering the monotonicity of f(x,y), we have $f(\mu_{B}(x_{i}),v_{B}(x_{i}))\leq f(\mu_{C}(x_{i}),v_{C}(x_{i}))$, $i\in \left\{1,2,\cdots ,n\right\}$. Under the conditions $a=\mu_{A}(x_{i})$, $b=v_{A}(x_{i})$, and $\forall i\in \left\{1,2,\cdots ,n\right\}$, the following expressions hold:

$$D^{I}(A,C)=\frac{1}{2n}\sum_{i=1}^{n}\sqrt{f(\mu_{C}(x_{i}),v_{C}(x_{i}))},\;D^{I}(A,B)=\frac{1}{2n}\sum_{i=1}^{n}\sqrt{f(\mu_{B}(x_{i}),v_{B}(x_{i}))}.$$

Therefore, we have $D^{I}(A,B)\leq D^{I}(A,C)$. (ii) In the conditions 0≤x≤a≤1 and 0≤b≤y≤1, we have ∂f/∂x≤0 and ∂f/∂y≥0. Thus, f(x,y) is a decreasing function of variable *x* and an increasing function of variable *y*. Setting $a=\mu_{C}(x_{i})$ and $b=v_{C}(x_{i})$, then $\mu_{A}(x_{i})\leq \mu_{B}(x_{i})\leq \mu_{C}(x_{i})=a$ and $v_{A}(x_{i})\geq v_{B}(x_{i})\geq v_{C}(x_{i})=b$, $i\in \left\{1,2,\cdots ,n\right\}$. Considering the monotonicity of f(x,y), we have $f(\mu_{B}(x_{i}),v_{B}(x_{i}))\leq f(\mu_{A}(x_{i}),v_{A}(x_{i}))$, $\forall i\in \left\{1,2,\cdots ,n\right\}$. For $a=\mu_{C}(x_{i})$, $b=v_{C}(x_{i})$, $\forall i\in \left\{1,2,\cdots ,n\right\}$, the following expressions hold:

$$D^{I}(A,C)=\frac{1}{2n}\sum_{i=1}^{n}\sqrt{f(\mu_{A}(x_{i}),v_{A}(x_{i}))},\;D^{I}(A,B)=\frac{1}{2n}\sum_{i=1}^{n}\sqrt{f(\mu_{B}(x_{i}),v_{B}(x_{i}))}.$$

Thus, we have $D^{I}(B,C)\leq D^{I}(A,C)$. Taking (i) and (ii) into account, we conclude that $D^{I}(A,B)\leq D^{I}(A,C)$ and $D^{I}(B,C)\leq D^{I}(A,C)$ in the condition of A⊆B⊆C. (4) The expression of $D^{I}(A,B)$ indicates that $D^{I}(A,B)\geq 0$ and $D^{I}(A,B)=0$ if A=B. Defining two AIFSs in $X=\{x_{1},x_{2},\cdots ,x_{n}\}$ as $F_{*}=\left\{\langle x,0,1\rangle \left|x\in X\right.\right\}$ and $F^{*}=\left\{\langle x,1,0\rangle \left|x\in X\right.\right\}$, for two AIFSs $A=\left\{\langle x,\mu_{A}(x),v_{A}(x)\rangle \left|x\in X\right.\right\}$ and $B=\left\{\langle x,\mu_{B}(x),v_{B}(x)\rangle \left|x\in X\right.\right\}$, we have $F_{*}\subseteq A\subseteq B\subseteq F^{*}$ based on the basic relation between AIFSs. The condition $F_{*}\subseteq A\subseteq B\subseteq F^{*}$ implies that $D^{I}(A,B)\leq D^{I}(F_{*},F^{*})$. Since AIFSs *A* and *B* are arbitrary, the relation $D^{I}(A,B)\leq D^{I}(F_{*},F^{*})$ holds in the set of all AIFSs defined in X. By Equation (9), the distance between $F_{*}$ and $F^{*}$ can be calculated as $D^{I}(F_{*},F^{*})=1$. Hence, $D^{I}(A,B)\leq 1$. From the above analysis, we obtain $0\leq D^{I}(A,B)\leq 1$. Therefore, the above analysis indicates that the distance $D^{I}(A,B)$ satisfies all axiomatic conditions of a distance measure, and thus $D^{I}(A,B)$ is a distance measure for AIFSs. □

## Appendix B

**Proof** **of** **Theorem** **3.** To be a knowledge measure of AIFSs, $K^{I}(A)$ defined in Equation (19) must satisfy all axiomatic properties defined in Definition 7. (KP1) Let *A* be a crisp set. We then have $\mu_{A}(x_{i})=1$, $v_{A}(x_{i})=0$ or $\mu_{A}(x_{i})=0$, $v_{A}(x_{i})=1$,i=1,2,⋯,n, which implies that $\mu_{A}(x_{i})+v_{A}(x_{i})=1$ and $\left|\mu_{A}(x_{i})-v_{A}(x_{i})\right|=1$. Thus, $K^{I}(A)=1$. In the condition of $0\leq \mu_{A}(x_{i})\leq 1$, $0\leq v_{A}(x_{i})\leq 1$, and $0\leq \mu_{A}(x_{i})+v_{A}(x_{i})\leq 1$, $K^{I}(A)=1$ can only be obtained in the case of $\mu_{A}(x_{i})+v_{A}(x_{i})=1$ and $\left|\mu_{A}(x_{i})-v_{A}(x_{i})\right|=1$, $\forall i\in \left\{1,2,\cdots ,n\right\}$. This indicates that $\forall i\in \left\{1,2,\cdots ,n\right\}$, $\mu_{A}(x_{i})=1$, $v_{A}(x_{i})=0$ or $\mu_{A}(x_{i})=0$, $v_{A}(x_{i})=1$, which means that *A* is a crisp set. Hence, $K^{I}(A)=1$ if and only if *A* is a crisp set. (KP2) In the case where $\forall i\in \left\{1,2,\cdots ,n\right\}$, $\pi_{A}(x_{i})=1$, we have $\mu_{A}(x_{i})=v_{A}(x_{i})=0$, $\forall i\in \left\{1,2,\cdots ,n\right\}$. Then, $K^{I}(A)=0$ can be obtained by Equation (19). The form of Equation (19) indicates that only in the case of ${\left(\mu_{A}(x_{i})-v_{A}(x_{i})\right)}^{2}=0$ and ${\left(\mu_{A}(x_{i})+v_{A}(x_{i})\right)}^{2}$ $\forall i\in \left\{1,2,\cdots ,n\right\}$ can we obtain $K^{I}(A)=0$. This implies that $\mu_{A}(x_{i})-v_{A}(x_{i})=0$ and $\mu_{A}(x_{i})+v_{A}(x_{i})=0$, $\forall i\in \left\{1,2,\cdots ,n\right\}$. Therefore, we have $\mu_{A}(x_{i})=v_{A}(x_{i})=0$, $\pi_{A}(x_{i})=1$, ∀i∈{1,2,⋯,n}. Thus, $K^{I}(A)$ complies with the property of KP2. (KP3) The expression of $K^{I}(A)$ can be rewritten as

$$K^{I}(A)=\frac{\sqrt{3}}{2n}\sum_{i=1}^{n}\sqrt{{\left|\mu_{A}(x_{i})-v_{A}(x_{i})\right|}^{2}+\frac{1}{3}{\left(1-\pi_{A}(x_{i})\right)}^{2}}.$$

It is explicit that $K^{I}(A)$ is monotonously increasing with $\left|\mu_{A}(x_{i})-v_{A}(x_{i})\right|$ if $\pi_{A}(x_{i})$ is fixed. By $0\leq \pi_{A}(x_{i})\leq 1$ and $0\leq 1-\pi_{A}(x_{i})\leq 1$, we can easily prove that $K^{I}(A)$ is monotonously decreasing with $\pi_{A}(x_{i})$ when $\left|\mu_{A}(x_{i})-v_{A}(x_{i})\right|$ remains unchanged. Then, $K^{I}(A)$ complies with the property of KP3. (KP4) By the definition of $A^{C}$, it is evident that $K^{I}(A^{C})=K^{I}(A)$. We note that $K^{I}(A)$ defined in Equation (19) complies with all properties in the axiomatic definition of a knowledge measure, so it is a knowledge measure for AIFSs. □
